# Osterix-Cre marks distinct subsets of CD45- and CD45+ stromal populations in extra-skeletal tumors with pro-tumorigenic characteristics

**DOI:** 10.7554/eLife.54659

**Published:** 2020-08-05

**Authors:** Biancamaria Ricci, Eric Tycksen, Hamza Celik, Jad I Belle, Francesca Fontana, Roberto Civitelli, Roberta Faccio

**Affiliations:** 1 Department of Orthopedics, Washington University School of MedicineSt. LouisUnited States; 2 Genome Technology Access Center, Department of Genetics, Washington University School of MedicineSt. LouisUnited States; 3 Department of Medicine, Division of Oncology, Washington University School of MedicineSt. LouisUnited States; 4 Department of Medicine, Division of Bone and Mineral Diseases, Washington University School of MedicineSt. LouisUnited States; 5 Shriners Children HospitalSt. LouisUnited States; UConn HealthUnited States; Maine Medical Center Research InstituteUnited States

**Keywords:** osterix, cancer, TME, CAF, bone, HSC, Mouse

## Abstract

Cancer-associated fibroblasts (CAFs) are a heterogeneous population of mesenchymal cells supporting tumor progression, whose origin remains to be fully elucidated. Osterix (Osx) is a marker of osteogenic differentiation, expressed in skeletal progenitor stem cells and bone-forming osteoblasts. We report *Osx* expression in CAFs and by using Osx-cre;TdTomato reporter mice we confirm the presence and pro-tumorigenic function of TdT^OSX^+ cells in extra-skeletal tumors. Surprisingly, only a minority of TdT^OSX^+ cells expresses fibroblast and osteogenic markers. The majority of TdT^OSX^+ cells express the hematopoietic marker CD45, have a genetic and phenotypic profile resembling that of tumor infiltrating myeloid and lymphoid populations, but with higher expression of lymphocytic immune suppressive genes. We find *Osx* transcript and Osx protein expression early during hematopoiesis, in subsets of hematopoietic stem cells and multipotent progenitor populations. Our results indicate that *Osx* marks distinct tumor promoting CD45- and CD45+ populations and challenge the dogma that Osx is expressed exclusively in cells of mesenchymal origin.

## Introduction

In the past couple of decades the common beliefs of how an incipient tumor grows and progresses to a metastatic stage have drastically changed due to the increasing findings of a tight crosstalk between tumor cells and the surrounding stroma, known as the tumor microenvironment (TME) (Hanahan and Weinberg, 2011). Tumor stroma comprises a variety of different cells from the mesenchymal and hematopoietic compartments, with pro- and anti-tumor functions. Cancer-associated fibroblasts (CAFs) are cells of the mesenchymal lineage involved in supporting various stages of tumorigenesis from growth to metastatic dissemination (Kalluri and Zeisberg, 2006; Chen and Song, 2019). Their abundance and phenotypic markers differ among the various types of cancers. CAFs support tumor growth by stimulating tumor cell proliferation, inhibiting apoptotic signals and anti-tumor immune responses, altering the extra-cellular matrix (ECM) to favor invasiveness, and by offering protection from chemotherapeutic approaches. Their heterogeneity probably accounts for the different tumor promoting effects and reflects the fact that CAFs can originate from multiple cellular sources (Chen and Song, 2019). Several studies indicate that CAFs derive from bone marrow mesenchymal stem and progenitor cells recruited at the tumor site. Additional evidence indicates that they can also derive from myofibroblasts, as well as from the trans-differentiation of local pericytes, endothelial and epithelial cells. In breast cancer, subsets of bone marrow-derived CAFs can exhibit a unique inflammatory profile depending on the location to which they are recruited, thus being functionally distinct from the resident CAFs and having better tumor-promoting functions (Raz et al., 2018). Adding more complexity to the origin of CAFs, an additional population expressing several markers commonly found in fibroblasts and CAFs, is the fibrocyte (Abe et al., 2001; McDonald and LaRue, 2012; van Deventer et al., 2013). Fibrocytes originate from the bone marrow, where they have been described to contribute to bone marrow fibrosis under pathological conditions (Ohishi et al., 2012). These cells have also been implicated in supporting tumor progression, but they differ from the bone marrow-derived CAF populations in that they also express the hematopoietic marker CD45, along with markers associated with the monocyte/macrophage lineage populations (Abe et al., 2001). Fibrocytes support tumor growth by making collagen, albeit at lower levels than the CAFs, releasing growth factors, commonly produced by immune suppressive myeloid cells, and by modulating resistance to anti-angiogenic therapy (Goto and Nishioka, 2017). Recent single-cell RNA sequencing (scRNAseq) studies further confirmed the complexity of the CAF populations, suggesting tumor specificity as well as functional differences among the various subsets (Bartoschek et al., 2018; Costa et al., 2018; Elyada et al., 2019). However, these studies did not directly address the origin of the various subsets, which could account for their specific cellular phenotype.

Because of their ability to produce collagen and other extracellular matrix proteins, we sought to determine whether a subset of bone marrow-derived CAFs could share markers of committed osteolineage cells. Osteolineage cells derive from bone marrow mesenchymal stem cells (MSCs) and their differentiation program is driven by sequential activation of two specific transcription factors, *Runx2* and *Sp7(Osx gene)*, and subsequent acquisition of a phenotype characterized by the ability to produce bone matrix, primarily type I collagen, and to mineralize (Nakashima et al., 2002). Although Osx is largely thought as a marker of differentiated osteoblasts, emerging data demonstrate that in the embryonic and perinatal bone marrow *Sp7* is expressed in definitive MSCs that give rise to the marrow stroma, including osteoblasts and adipocytes (Liu et al., 2013; Mizoguchi et al., 2014). Furthermore, during embryogenesis, Osx is present in extra-skeletal tissues, including the olfactory bulb, the intestine and the kidney (Chen et al., 2014; Jia et al., 2015).

Based on the above observations, we hypothesized that a subset of CAFs, derived from Osx+ cells in the bone marrow, contributes to ECM (i.e. collagen) production at the tumor site, thereby creating a tumor supporting stroma. Using a cell tracking system, we found the presence of cells targeted by the Osx promoter within the TME; these TdT^OSX^+ cells favor tumor growth when co-injected with tumor cells in mice. Surprisingly, only a minority of tumor-resident cells derived from Osx+ cells expresses fibroblast markers, extracellular matrix and matrix remodeling genes. The majority of these newly identified TdT^OSX^+ tumor infiltrating cells are also positive for CD45, a marker of hematopoietic lineage, and share markers expressed by tumor-infiltrating immune cells. Importantly, we confirmed *Sp7* transcripts and Osx protein in a subset of hematopoietic stem cells (HSC), giving rise to TdT^OSX^+;CD45+ tumor infiltrating immune populations. This study further identifies new populations of TME cells targeted by Osx and challenges the use of Osx-cre driven lineage tracing mouse models to exclusively study mesenchymal lineage cell fate.

## Results

### Embryonic and adult-derived osteolineage Osx+ cells are present in extra-skeletal tumors

**Figure 1.**
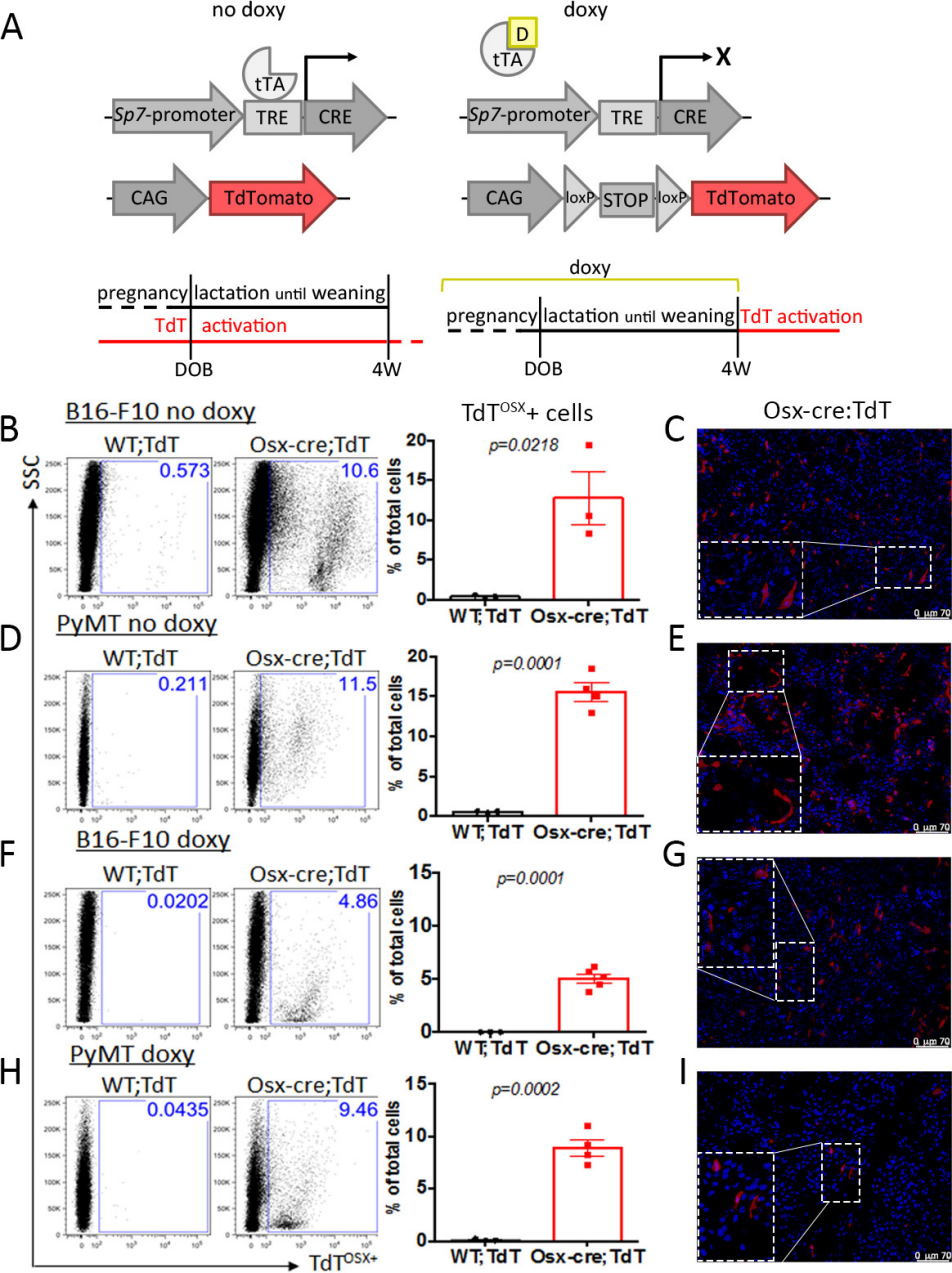
Embryonic and adult-derived Osx+ cells are present in primary tumors at extra-skeletal sites. (**A**) Doxycycline (doxy)-repressible Sp7-cre/loxP mouse model used to activate Ai9/TdTomato expression for lineage tracing experiments. In no doxy-fed mice, TdT is expressed in embryonic-derived osteolineage cells (left), while in mice fed a doxy diet until weaning, TdT is expressed in adult-derived osteolineage cells. (**B–I**) Flow cytometry analysis and fluorescence images of primary tumors showing presence of TdT^OSX^+ cells in no-doxy fed Osx-cre;TdT mice or WT;TdT controls inoculated with B16-F10 melanoma subcutaneously (**B–C**), or with PyMT breast cancer cells in the mammary fat pad (MFP) (**D–E**), and in doxy-fed mice injected with B16-F10 subcutaneously (**F–G**), or with PyMT in the MFP (**H–I**). Slides for fluorescence images were counterstained with DAPI (blue), magnification 200X. Inserts are further magnified 4.5 folds. Data representative of a single experiment, experiments were repeated between 2 and 5 times. p values represent Student t-test statistical analysis. Figure 1—source data 1.Relates to FACS analysis in panels B, D, F, H.

To determine whether osteolineage cells may be present in the TME, we crossed the established tetracycline-dependent *Tg(Sp7-tTA,tetO-EGFP/cre)1Amc/J* (Osx-cre) to the *B6.Cg-Gt(ROSA)26Sortm9(CAG-tdTomato)Hze/J* (TdT) to generate the Osx-cre;TdT reporter mouse model (Rodda and McMahon, 2006). When constitutively activated, TdT marks the entire osteolineage, including bone surface osteoblasts, osteocytes and bone marrow cells with mesenchymal stem and osteoprogenitor cell features; while delaying Osx-cre expression until postnatally restricts TdT targeting to committed osteoblasts and osteocytes (Mizoguchi et al., 2014; Fontana et al., 2017). Therefore, Osx-cre;TdT mice and control animals carrying only the TdT transgene (WT;TdT) were kept on standard chow to allow constitutive embryonic transgene activation, or fed a doxycycline (doxy)-containing diet until weaning to suppress transgene activation until one month of age (Figure 1A). We previously reported that doxy-fed mice display less than 1% of spontaneous recombination in the bone residing osteoblasts at weaning, but full transgene activation 1 month thereafter (Fontana et al., 2017).

**Figure 1—figure supplement 1.**
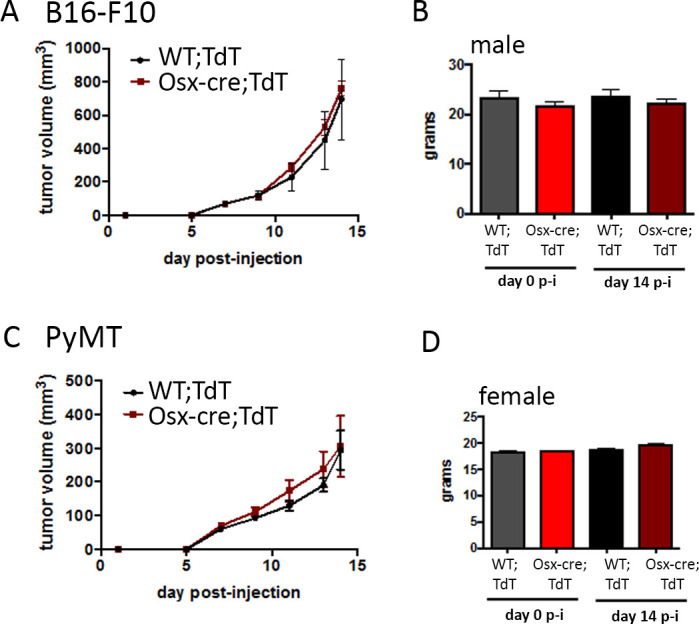
No difference in tumor growth and body weight between sex and age matched cre positive (Osx-cre;TdT) and negative (WT;TdT) mice. . (**A**) Tumor growth in Osx-cre;TdT and WT;TdT male mice injected subcutaneously with B16-F10 tumor cells. (**B**) Body weight of the mice showed in (**A**) at the day of tumor injection (day 0) and at the end point (day 14 post-injection). (**C**) Tumor growth in Osx-cre;TdT and WT;TdT female mice injected in the MFP with PyMT tumor cells. (**D**) Body weight of the mice showed in (**C**) on day 0 and day 14 post-injection.

Osx-cre;TdT reporter mice and WT;TdT fed a normal diet (no doxy) were inoculated with either 10^5^ B16-F10 melanoma cells subcutaneously or 10^5^ PyMT breast cancer cells in the mammary fat pad (MFP). To account for the possible growth delay sometimes observed in no-doxy fed Osx-cre mice (Davey et al., 2012), tumor cells were injected at 12 weeks of age, when the growth delay is fully recovered. Importantly, no differences in tumor growth were observed in age and sex matched Osx-cre;TdT and WT;TdT mice, excluding any potential confounding effect of the Osx-cre (Figure 1—figure supplement 1). To assess the presence of osteolineage Osx+ derived cells within the tumor stroma of no doxy-fed mice, we performed flow cytometry analysis on B16-F10 and PyMT tumors isolated 2 weeks post-inoculation, and found that about 10–18% of the total cells were TdT^OSX^+ (Figure 1B and D). Histological analysis of frozen sections from the same tumors confirmed the presence of TdT^OSX^+ cells. These cells had either round or elongated shape, indicating morphologic heterogeneity within the TdT^OSX^+ population (Figure 1C and E). These TdT^OSX^+ cells within the tumor stroma of no-doxy mice may derive from resident Osx-expressing embryonic progenitors or from mobilization of bone marrow Osx+ cells.

Remarkably, TdT^OSX^+ cells were also present in the B16-F10 and PyMT TME of doxy-fed animals 4 weeks post weaning, although their numbers were lower than in tumors isolated from no doxy-treated mice (Figure 1F and H). Fluorescence micrographs also confirmed the presence of TdT^OSX^+ cells in the stroma in both tumor models (Figure 1G and I), with the same pleiotropic morphology as seen in no doxy-treated mice. Thus, embryonic and adult-derived osteolineage Osx+ cells are present in the stroma of extra-skeletal tumors. Based on these findings, the doxy-fed Osx-cre;TdT mice were used for all the subsequent studies.

### TdT^OSX^+ cells from primary tumors express mesenchymal markers

**Figure 2.**
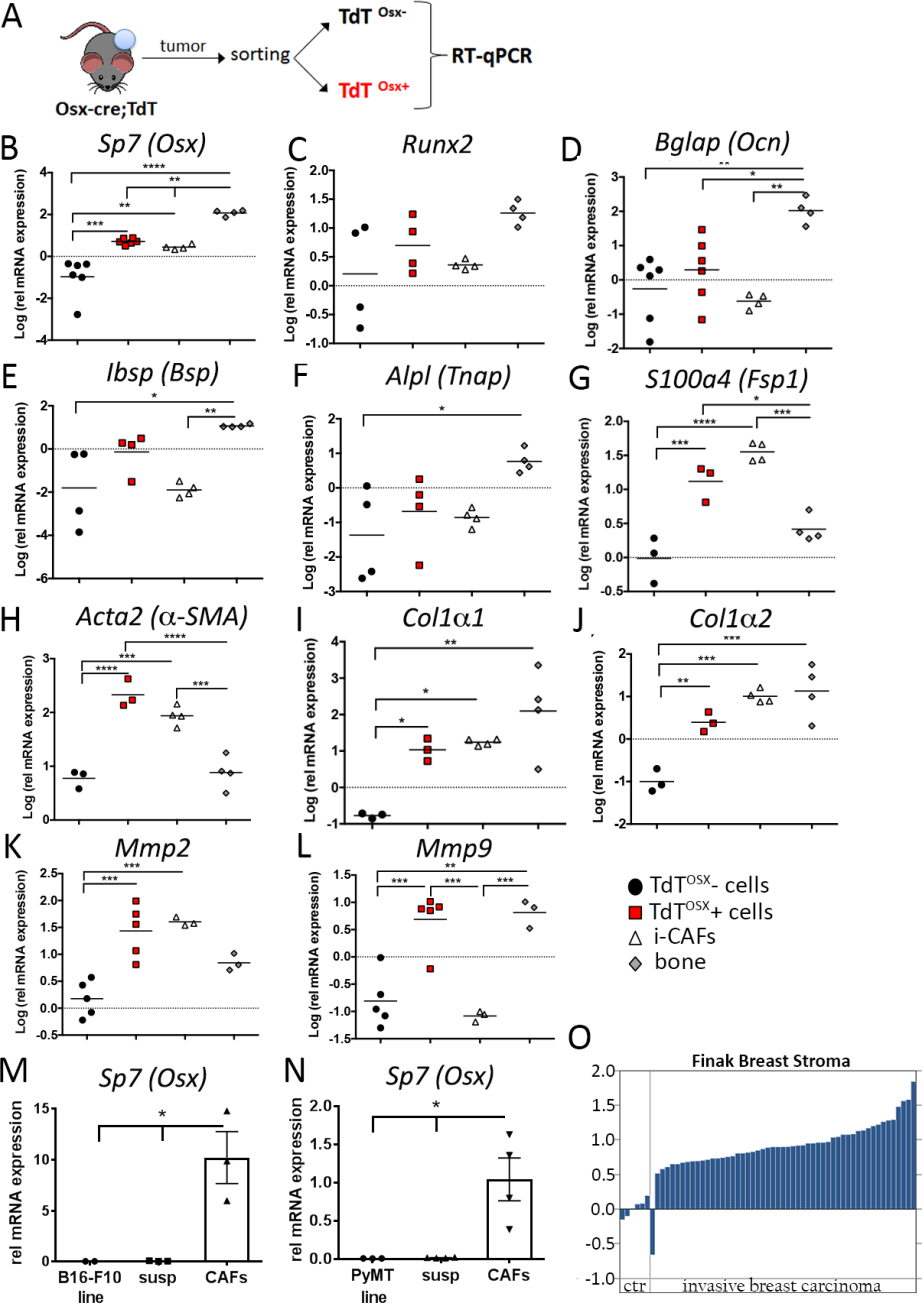
TdT^OSX^+ cells from primary tumors express mesenchymal markers. (**A**) Experimental design for isolating TdT^OSX^+ cells from primary tumors isolated from doxy-fed Osx-cre;TdT mice inoculated with B16-F10. TdT^OSX^- cells comprise all TdT negative cells including the tumor cells. Immortalized CAFs (iCAFs) and crushed long bones were used as reference controls. (**B–L**) Quantitative Real-Time PCR for (**B**) *Sp7(Osterix)*, (**C**) *Runx2*, (**D**) *Bglap(Osteocalcin)*, (**E**) *Ibsp (Bone sialoprotein)*, (**F**) *Alpl (Tnap, Tissue non-specific alkaline phosphatase)*, (**G**) *S100a4 (Fsp1),* (**H**) *Acta2 (α-SMA)* (**I**) *Collagen1a1* (**J**) *Collagen1a2,* (**K**) *Mmp2 (Metalloproteinase2)* and (**L**) *Mmp9 (Metalloproteinase9)* genes in TdT^OSX^+, TdT^OSX^- cells, iCAFs and bone. Each data point includes cells isolated from 3 to 4 mice and from two independent experiments. (**M–N**) Real-Time PCR of *Sp7(Osterix)* in B16-F10 or PyMT cell lines, CAFs isolated from primary B16-F10 or PyMT tumors or from the remaining cells comprising the tumor minus the CAFs (susp) (n = 3–4 mice). (**O**) *Sp7(Osx)* expression based on the Finak Breast Cancer Stroma gene set in the Oncomine cancer microarray database. Significance was determined by one-way ANOVA with Tukey post-hoc test, *p<0.05, **p<0.01, ***p<0.001, ****p<0.0001. Figure 2—source data 1.Relates to Real-Time PCR data.

To determine the phenotype of TdT^OSX^+ cells more in depth, we isolated TdT^OSX^+ cells by FACS sorting 14 days after inoculation of B16-F10 tumor cells into doxy-fed Osx-cre;TdT reporter mice (Figure 2A). Due to the limited number of TdT^OSX^+, tumors from 2 to 4 mice were pooled for FACS sorting and reported as one experimental replicate (single data point). Messenger RNA from an immortalized murine CAF cell line (i-CAFs) and cortical long bone devoid of bone marrow cells were used as controls. Real-Time PCR confirmed *Sp7 e*xpression in TdT^OSX^+ cells but not in the TdT^OSX^– fraction, which includes the remaining stroma and the tumor cells (Figure 2B). Interestingly, *Sp7* expression was also detected in i-CAFs (Figure 2B), and as expected in bone extracts in higher abundance. Expression of osteoblast-specific markers such as *Runx2*, *Bglap (Ocn)* (Osteocalcin), *Ibsp (Bsp, bone sialoprotein)* and *Alpl (Tnap, tissue non-specific alkaline phosphatase)* were negligible in TdT^OSX^+ cells and undetectable or barely detectable in the i-CAFs (Figure 2C–F). However, *S100a4(Fsp1)* (Fibroblast-specific protein 1) and *Acta2 (α-SMA)* (α-smooth muscle actin), two CAFs specific markers, were detected in TdT^OSX^+ cells (Figure 2G and H). TdT^OSX^+ also expressed *Col1a1* (Collagen Type Ia1) and *Col1a2* (Collagen Type Ia2) similar to i-CAFs and bone extracts (Figure 2I and J). Expression of matrix metalloproteinases, *Mmp2* and *Mmp9*, involved in matrix remodeling and highly expressed in CAFs and osteoblasts respectively, was also detected in the TdT^OSX^+ cells (Figure 2K and L).

To validate expression of *Sp7* in CAFs from primary tumors, we orthotopically injected B16-F10 or PyMT cells in 8 week old WT mice and the CAFs were isolated as the adherent fraction of a single cell suspension of the tumor mass. We detected *Sp7* expression only in CAFs but not in the tumor cell lines nor in the non-adherent fraction of the single cell suspension from the tumor mass (Figure 2M and N). Importantly, corroborating our findings in mice, using Oncomine (Rhodes et al., 2004) we found that microarray analysis of human breast stromal cells collected from patients with invasive breast carcinoma from the Finak gene set (Finak et al., 2008) showed significantly higher *Sp7* expression in the tumor associated stroma compared to the adjacent normal breast tissue (Figure 2O). Thus, a subset of cells in the TME of mice and humans express *Sp7*, an osteolineage cell marker, but also markers associated with the CAFs. TdT^OSX^+ cells may represent a subpopulation of CAFs with specific features.

### Tumor resident but not bone marrow resident TdT^OSX^+ cells increase tumor growth

**Figure 3.**
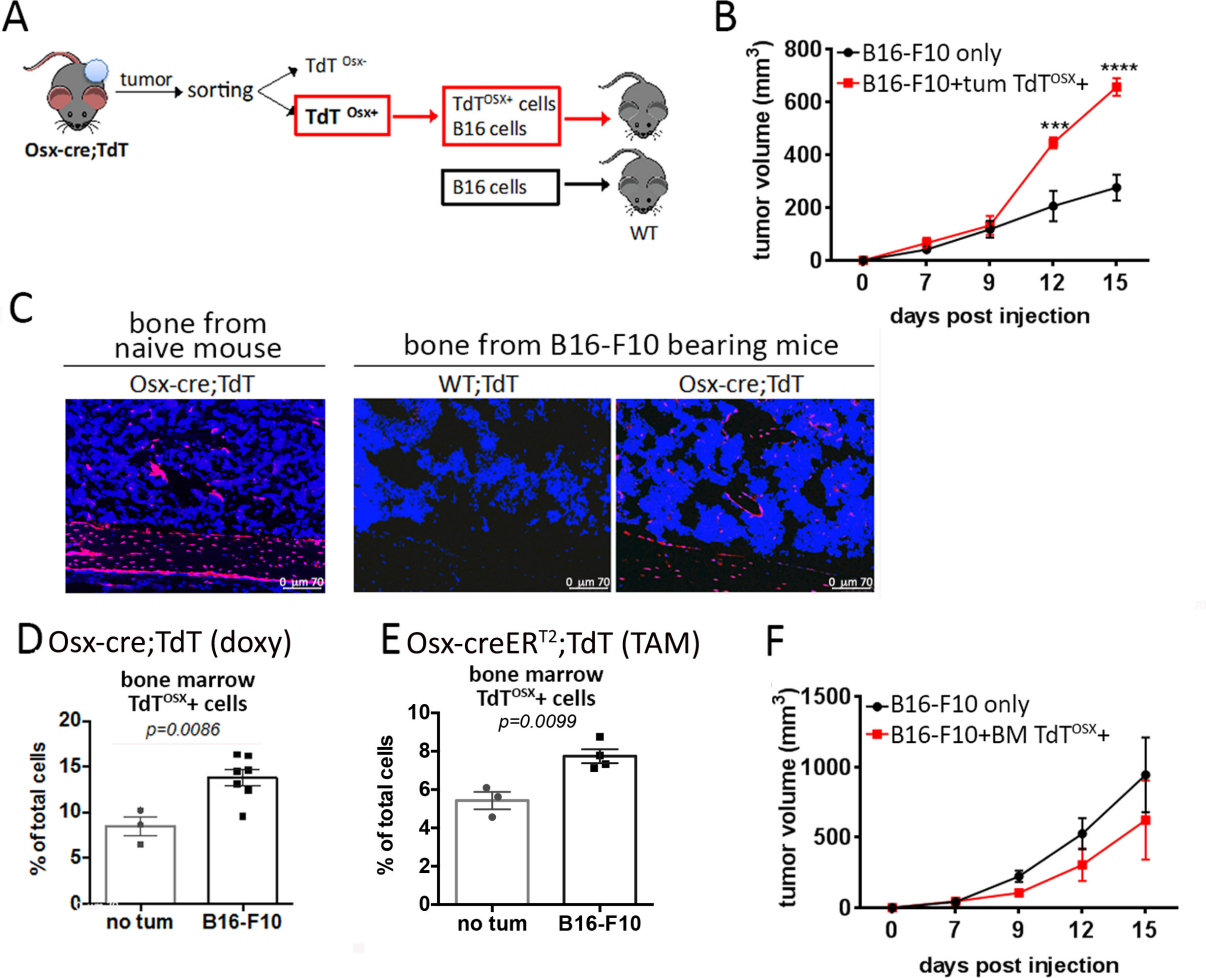
Tumor resident but not bone marrow resident TdT^OSX^+ cells increase tumor growth. (**A**) Model showing isolation of TdT^OSX^+ cells from B16-F10 primary tumors injected into doxy-fed Osx-cre;TdT mice and re-inoculation of TdT^OSX^+ cells together with B16-F10 tumor cells into WT recipient mice at the ratio 5:1. Mice injected with B16-F10 alone were used as controls. (**B**) Tumor growth of experimental model described in (**A**) determined by caliper measurement. n = 3/group, experiment repeated twice. Significance was determined by one-way ANOVA with Tukey post-hoc test. (**C**) Fluorescence images of bone sections showing presence of TdT^OSX^+ within the bone and bone marrow of naïve Osx-cre;TdT, Osx-cre;TdT mice inoculated subcutaneously with B16-F10 tumors or WT;TdT mice used as negative control. Sections were counterstained with DAPI (blue), magnification 200X. (**D–E**) Quantification of TdT^OSX^+ cells in the bone marrow of tumor-free and B16-F10 tumor bearing (**D**) Osx-cre;TdT mice (doxy-fed) and (**E**) Osx-creER^T2^;TdT (TAM-treated) determined by FACS. Experiment repeated twice. Significance was determined by student t-test statistical analysis. (**F**) Tumor growth of WT mice inoculated with bone marrow-derived TdT^OSX^+ cells from B16-F10 bearing doxy-fed Osx-cre;TdT mice together with B16-F10 tumor cells (ratio 5:1). Mice injected with B16-F10 alone were used as controls. n = 3/group. Significance was determined by two-way ANOVA followed by Tukey post-hoc test. ***p<0.001, ****p<0.0001.

To determine whether tumor resident TdT^OSX^+ cells can affect tumor growth as do CAFs, TdT^OSX^+ cells were sorted from primary B16-F10 tumors inoculated in 8 week old doxy-fed Osx-cre;TdT mice and re-injected along with B16-F10 tumor cells at 5:1 ratio into age-matched WT recipient animals (Figure 3A). Remarkably, co-injecting TdT^OSX^+ with tumor cells resulted in larger tumors (Figure 3B) compared to injection of B16-F10 cells alone.

**Figure 3—figure supplement 1.**
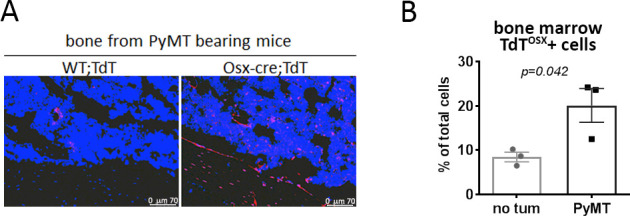
TdT^OSX^+ cells increase in the bone marrow of PyMT tumor bearing mice. (**A**) Fluorescence images of WT;TdT and Osx-cre;TdT mice inoculated with PyMT tumors in the MFP. Slides for fluorescence images were counterstained with DAPI (blue), magnification 200X. (**B**) Quantification of TdT^OSX^+ cells in the bone marrow of tumor-free and PyMT bearing mice. Experiment repeated twice. p value represents Student t-test statistical analysis. Figure 3—figure supplement 1—source data 1.Relates to FACS analysis.

**Figure 3—figure supplement 2.**
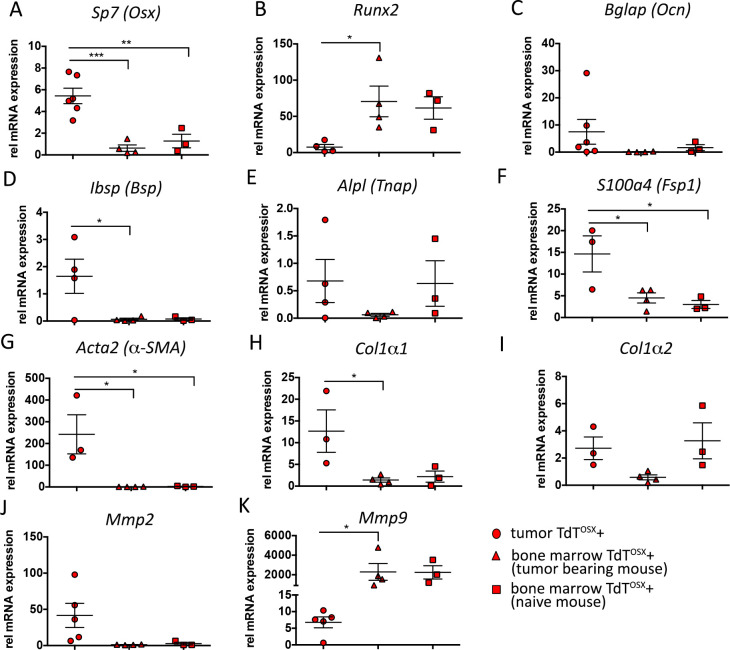
Differential expression of mesenchymal markers in TdT^OSX^+ cells isolated from tumor site or bone marrow. (**A–K**) Quantitative Real-Time PCR for (**A**) *Sp7 (Osterix)*, (**B**) *Runx2*, (**C**) *Bglap (Osteocalcin)*, (**D**) *Ibsp (Bone sialoprotein)*, (**E**) *Alpl (Tnap, Tissue non-specific alkaline phosphatase)*, (**F**) *S100a4 (Fsp1),* (**G**) *Acta2 (α-SMA),* (**H**) *Collagen 1a1*, (**I**) *Collagen 1a2,* (**J**) *Mmp2 (Metalloproteinase2)* and (**K**) *Mmp9 (Metalloproteinase9)* genes in TdT^OSX^+ isolated from the B16-F10 melanoma primary tumors, bone marrow of B16-F10 bearing mice and bone marrow of naïve tumor-free mice. Figure 3—figure supplement 2—source data 1.Relates to Real-Time PCR.

Because TdT^OSX^+ cells normally reside in the bone microenvironment (Fontana et al., 2017; Strecker et al., 2013), we next examined bone sections from 8 week old doxy-fed Osx-cre;TdT reporter mice, naïve or bearing soft tissue B16-F10 or PyMT tumors. We confirmed presence of TdT^OSX^+ cells on the surface of cortical and trabecular bone, TdT^OSX^+ osteocytes, and also abundant staining in the bone marrow. TdT was not detected WT;TdT mice (Figure 3C and Figure 3—figure supplement 1A). We further analyzed the percentage of TdT^OSX^+ cells in the bone marrow by FACS and found that TdT^OSX^+ cells accounted for about 10% of total marrow cells in naïve mice and their number increased in mice injected with B16-F10 or PyMT tumor cells (Figure 3D and Figure 3—figure supplement 2B). Similar findings were observed in the Tamoxifen (TAM) inducible *Sp7-creER^T2^; Rosa26^<fs-TdTomato>^* (Osx-creER^T2^;TdT) mouse model. TAM was administered to 7 week old mice via IP injections starting 3 days prior to B16-F10 tumor inoculation, and 1, 6, and 9 days post tumor cell injection (Figure 4—figure supplement 1A). No tumor bearing mice were used as control. Similar to the Tet-OFF Osx-cre model, we found that bone marrow TdT^OSX^+ cells significantly increased in presence of a tumor (Figure 3E).

To test their capacity to promote tumor growth, bone marrow-derived TdT^OSX^+ cells were isolated from tumor bearing mice and re-injected with B16-F10 tumor cells into WT mice (5:1 ratio). In contrast to what observed with tumor-derived TdT^OSX^+ cells, bone marrow-derived TdT^OSX^+ cells did not enhance tumor growth compared to mice injected with tumor cells alone; if anything, there was a trend towards a smaller tumor size (Figure 3F). This result further prompted us to isolate bone marrow-derived TdT^OSX^+ cells and analyze the expression levels of the same mesenchymal genes analyzed in the tumor-derived TdT^OSX^+ cells (Figure 2). Interestingly, TdT^OSX^+ cells from the TME expressed higher levels of *Sp7*, CAF markers *S100a4* and *Acta2*, and matrix proteins *Col1a1* and *Ibsp* compared to TdT^OSX^+ from the bone marrow (Figure 3—figure supplement 2). Bone marrow TdT^OSX^+ cells instead expressed significantly higher levels of the early osteoblast transcription factor *Runx2* and of the protease *Mmp9*, while no differences in other osteoblast markers such as *Bglap* and *Alpl* were detected between the tumor and bone marrow sorted TdT^OSX^+ cells (Figure 3—figure supplement 2).

These results suggest that Osx+ cells infiltrating a tumor are functionally and phenotypically distinct from bone marrow resident Osx+ cells.

### The majority of TdT^OSX^+ cells express CD45

**Figure 4.**
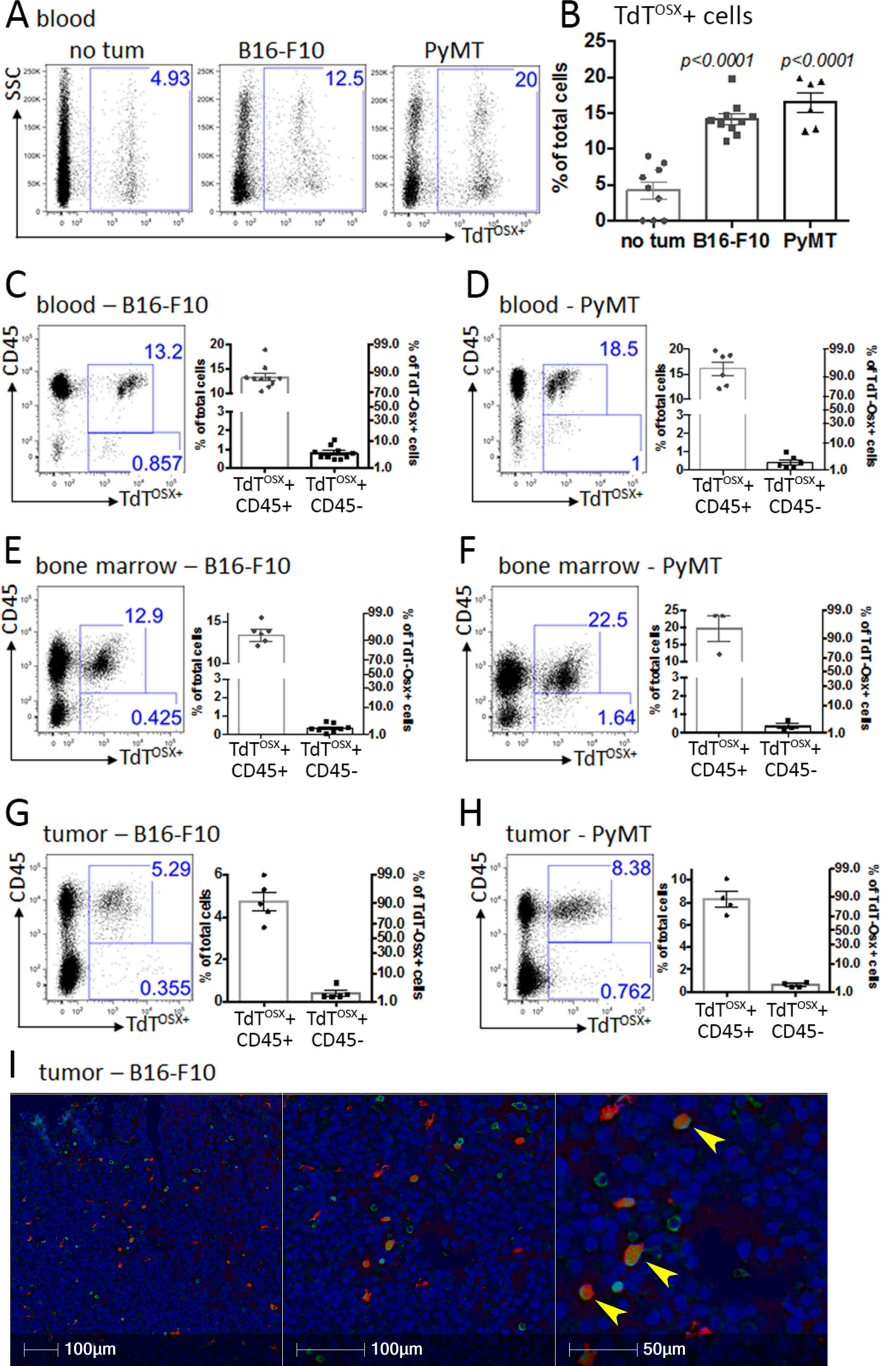
TdT^OSX^+ cells express the immune surface marker CD45. (**A–B**) Representative FACS dot plots and quantification of TdT^OSX^+ cells in the peripheral blood of tumor-free and B16-F10 or PyMT tumor bearing mice. Significance was determined by student t-test statistical analysis for each tumor model relative to no tumor controls. (**C–D**) Representative FACS dot plots and quantification of TdT^OSX^+;CD45+ and TdT^OSX^+;CD45- populations in the blood of doxy-fed Osx-cre;TdT mice injected with B16-F10 or PyMT cells. (**E–H**) Representative FACS dot plots and quantification of TdT^OSX^+;CD45+ and TdT^OSX^+;CD45- populations in the bone marrow (**E–F**) or tumor site (**G–H**) of doxy-fed Osx-cre;TdT mice injected with B16-F10 or PyMT cells, respectively. Experiments were repeated at least twice. (**I**) Immunohistochemistry staining of paraffin-embedded B16-F10 tumors inoculated into Osx-cre;TdT reporter mice, pseudo colored with red representing TdT^OSX^+ cells (RFP stained), green representing CD45+ cells (DAB stained) and blue representing nuclei (hematoxylin), magnification 200X. Figure 4—source data 1.Relates to FACS analysis in panels B, C, D, E, F, G, H.

To gain insights on the possible origin of tumor-derived TdT^OSX^+ cells, we performed FACS analysis of circulating cells in naïve mice and two weeks post B16-F10 or PyMT tumor inoculation. Interestingly, we found 5–8% of TdT^OSX^+ cells in the blood of tumor-free doxy-fed Osx-cre;TdT mice, and this percentage was 3- to 4-fold higher in tumor-bearing animals (Figure 4A and B). The unexpected high number of circulating TdT^OSX^+ cells prompted us to determine whether they may express the immune marker CD45, which is also present in fibrocytes. Surprisingly, 95% of TdT^OSX^+ cells in circulation of tumor bearing mice were also positive for CD45, and they represented about 13–18% of total blood cells (Figure 4C and D). Similarly, about 92–95% of total TdT^OSX^+ cells in the bone marrow were CD45+ (Figure 4E and F), representing 12–20% of total marrow cells. Such finding explained the low expression levels of mesenchymal markers in the bone marrow-derived TdT^OSX^+ cells (Figure 3—figure supplement 2). Intriguingly, also the majority of TdT^OSX^+ cells in the TME expressed CD45 (Figure 4G and H), data further confirmed by RT-PCR in TdT^OSX^+ fraction sorted from B16-F10 tumors (not shown).

**Figure 4—figure supplement 1.**
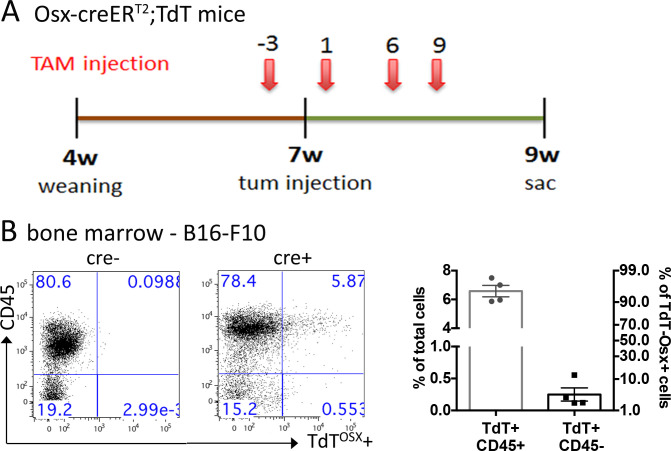
Presence of TdT^OSX^+ cells in bone marrow (BM) of TAM-pulsed Osx-creER^T2^;TdT reporter mice. (**A**) Schematic representation of TAM administration and tumor injection in Osx-creER^T2^;TdT mice. (**B**) Representative image of flow cytometry dot plots and quantification of TdT^OSX^+;CD45+ and TdT^OSX^+;CD45- populations in the bone marrow of B16-F10 bearing mice. A cre negative mouse was used as control to set gates. Experiment was repeated three times. Figure 4—figure supplement 1—source data 1.Relates to FACS analysis.

**Figure 4—figure supplement 2.**
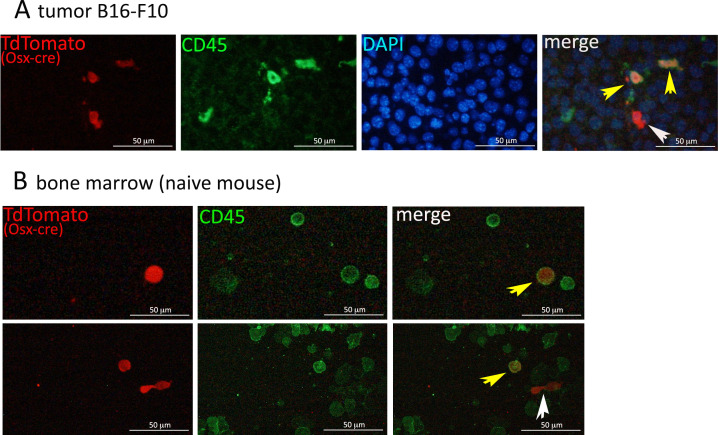
Co-expression of Osx-driven TdTomato and the immune marker CD45. (**A**) Immunofluorescence on paraffin-embedded B16-F10 tumors isolated from Osx-cre;TdT reporter mice. Staining was done using anti-RFP (red) to detect TdTomato and anti-CD45 (green) to stain immune populations. Slides were counterstained with DAPI (blue), magnification 200X. (**B**) Staining for CD45 on a single cell suspension from bone marrow of Osx-cre;TdT reporter mice, magnification 200X. Yellow arrows point to TdT^OSX^+ single cells expressing CD45, while the white arrow points to a TdT^OSX^+ cell negative for CD45.

To exclude a possible off-target effect of the doxy-dependent Tet-OFF system, we turned to the TAM inducible Osx-creER^T2^;TdT model. FACS analysis confirmed presence of both CD45- and CD45+ TdT^OSX^+ populations in the bone marrow of B16-F10 bearing Osx-creER^T2^;TdT mice, but not in the cre negative mice (Figure 4—figure supplement 1B).

Next, to confirm that expression of CD45 in the TdT^OSX^+ cells was not due a tight interaction between a CD45+ immune cell and TdT^OSX^+ mesenchymal cell, we performed CD45 immunostaining on paraffin-embedded B16-F10 tumor sections and on bone marrow single cell suspension from Osx-cre;TdT reporter mice. We found co-localization of Osx-driven TdTomato and CD45 in the same cell, confirming that CD45 is indeed expressed in subsets of TdT^OSX^+ cells (Figure 4I and Figure 4—figure supplement 2). Thus, Osx not only marks mesenchymal cells, but also cells expressing the hematopoietic marker CD45, suggesting that they could represent bone marrow fibrocytes or an immune cell subset.

### Presence of functionally distinct populations of TdT^OSX^+ cells in the tumor microenvironment

To further characterize the phenotype of the CD45- and CD45+ TdT^OSX^+ populations, we next performed RNAseq analysis. We subcutaneously injected the GFP-labeled PyMT-BO1-tumor line in Osx-cre;TdT reporter mice. Two weeks post tumor inoculation, the stromal cells were separated from the GFP+ tumor cells and sorted based on the expression, or lack of thereof, of TdT and CD45 markers. We sorted 4 groups of cells: double negative (TdT^OSX^-;CD45-), representing the non-immune tumor stroma, CD45 single positive (TdT^OSX^-;CD45+), representing the tumor infiltrating immune populations, TdT^OSX^ single positive (TdT^OSX^+;CD45-), and double positive (TdT^OSX^+;CD45+).

**Figure 5.**
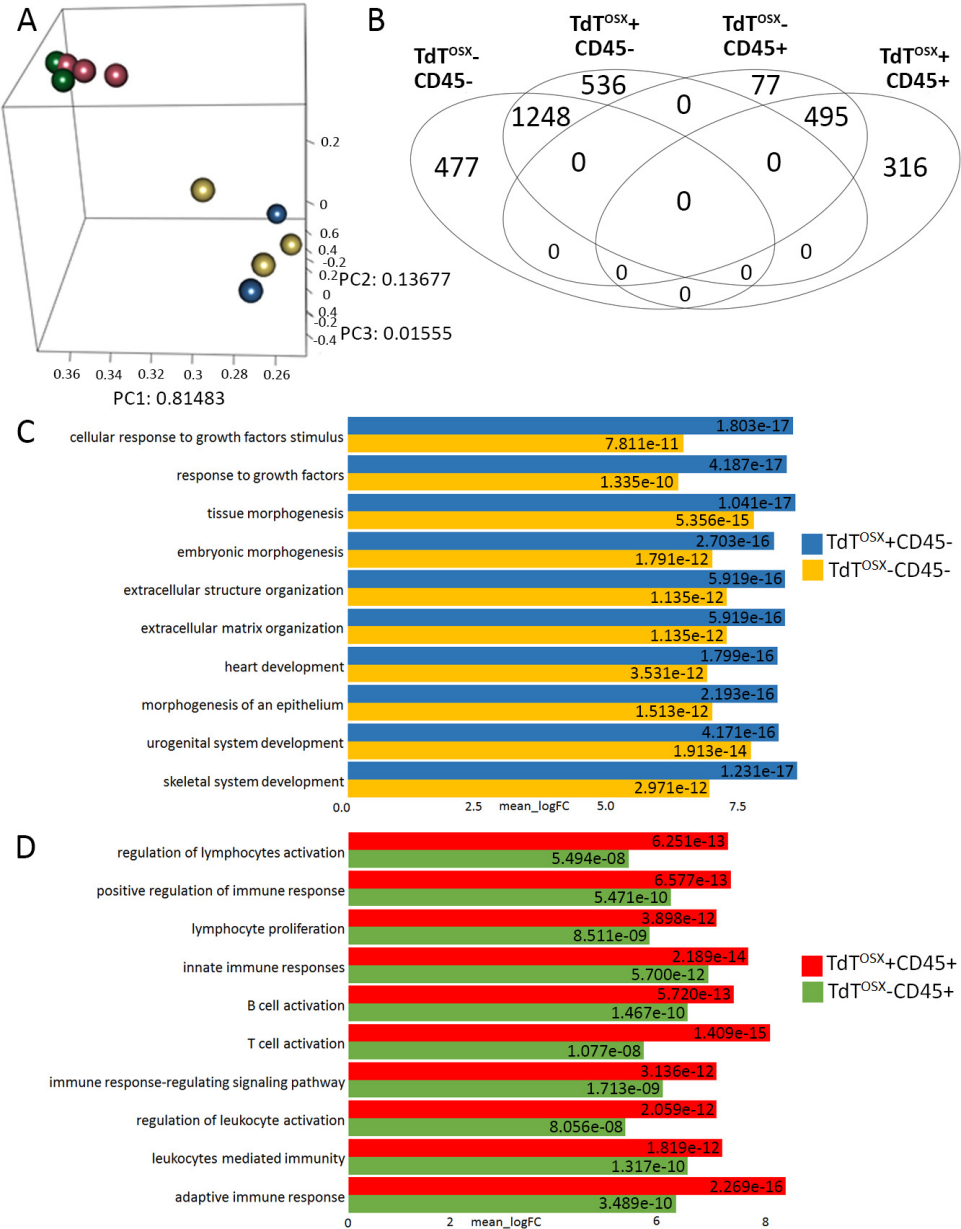
TdT^OSX^+;CD45- and TdT^OSX^+;CD45+ are two functionally distinct populations in the tumor microenvironment. (**A**) Multidimensional Principal Component Analysis of RNAseq data obtained from four tumor stroma subsets sorted according to TdT^OSX^ and CD45 expression. (**B**) Venn diagram depicting uniquely and commonly expressed genes among the four groups. (**C–D**) First ten GO pathways obtained from the GO analysis of the log two fold-changes for (**C**) CD45 negative subsets and (**D**) CD45 positive subsets.

Principal components analysis of RNA-seq expression patterns across all four groups revealed clustering that was uniquely dependent on CD45 expression and suggested that there were only two very distinct cell populations regardless of TdT^OSX^ (Figure 5A). Inspection of each cell population in a one versus all other approach for only statistically significant up-regulated genes (FDR >= 0.05, log2 fold-change >= 2) to identify robustly expressed biomarkers, showed that all four groups shared many genes in common based on CD45 expression alone. The CD45 negative populations shared 1248 genes in common with 536 in the TdT^OSX^ single positive and 477 in the double negative cells uniquely up-regulated (Figure 5B). Likewise, the CD45 single and double positive cells shared 495 significantly up-regulated genes with an additional 77 and 316 uniquely expressed, respectively.

**Figure 5—figure supplement 1.**
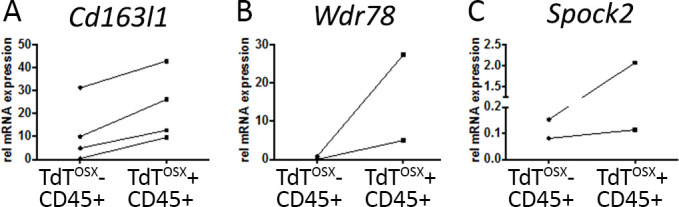
Validation of RNAseq data. Real Time PCR performed on TdT^OSX^+;CD45+ and TdT^OSX^+;CD45- populations sorted from B16-F10 tumors in doxy-fed mice. Each sample is a pool of two tumors. Data show three genes (**A**) *Cd163l1*, (**B**) *Wdr79* and (**C**) *Spock2* to be upregulated in the TdT^OSX^+;CD45+ compared to TdT^OSX^-;CD45+ cells. Figure 5—figure supplement 1—source data 1.Relates to Real-Time PCR data.

Subsequent Gene Ontology (GO) analysis of the log_2_ fold-changes for each cell population revealed that the CD45 negative subsets showed upregulation of GO pathways related to cellular responses to growth factors, extracellular structure and matrix organization and skeletal system development, confirming their mesenchymal nature (Figure 5C). Among the top upregulated genes in the TdT^OSX^ single positive cells versus the double negative (Table 1) we found several markers expressed by osteolinage cells, such as fibromodulin (*Fmod*), a binding protein regulating bone mineralization (Gori et al., 2001) and also involved in TGF*β* signaling during cancer pathogenesis (Pourhanifeh et al., 2019), Collagen 24a1 (*Col24a1*), known to modulate collagen chain trimerization (Koch et al., 2003; Matsuo et al., 2006), *Frem1*, a protein involved in the formation and organization of basement membranes (Vissers et al., 2011), and *Msx1*, a transcription factor important in craniofacial bone development (Orestes-Cardoso et al., 2002). TdT^OSX^ single positive cells also expressed high levels of fibulin 7 (*Fbln7*), a cell adhesion molecule overexpressed in glioblastoma by pericytes and involved in neovascularization (de Vega et al., 2019; Ikeuchi et al., 2018). Such result confirmed the osteolineage nature of the TdT^OSX^+ cells and indicated their tumor supporting role.

**Table 1.** Top 50 uniquely up-regulated genes in tumor-derived TdT^OSX^+;CD45- cells. List of the 50 most highly expressed genes in the TdT^OSX^+;CD45- cells compared to the TdT^OSX^-;CD45- cells isolated from the TME.

external_gene_name	description	TdTOSX+CD45- logFC	TdTOSX+CD45- linearFC
Reg1	regenerating islet-derived 1	7.675801	204.477899
Fbln7	fibulin 7	7.184048	145.416614
Adcyap1r1	adenylate cyclase activating polypeptide 1 receptor 1	6.864422	116.51905
Col24a1	collagen, type XXIV, alpha 1	6.788179	110.521192
Moxd1	monooxygenase, DBH-like 1	6.74623	107.35382
Fmod	fibromodulin	6.739645	106.864989
4930562D21Rik	RIKEN cDNA 4930562D21 gene	6.149924	71.008705
Angpt4	angiopoietin 4	6.131627	70.113799
Tmem132c	transmembrane protein 132C	5.933002	61.095844
Frem1	Fras1 related extracellular matrix protein 1	5.906166	59.969876
Cxcl5	chemokine (C-X-C motif) ligand 5	5.88881	59.252728
Ccl19	chemokine (C-C motif) ligand 19	5.748355	53.756054
Rab15	RAB15, member RAS oncogene family	5.696057	51.842269
Prlr	prolactin receptor	5.673651	51.04334
Msx1	msh homeobox 1	5.657661	50.480738
Pabpc4l	poly(A) binding protein, cytoplasmic 4-like	5.657301	50.46814
Reg3g	regenerating islet-derived 3 gamma	5.651841	50.2775
Rflna	refilin A	5.651765	50.274843
Adamts3	a disintegrin-like and metallopeptidase (reprolysin type) with thrombospondin type 1 motif, 3	5.52646	46.092506
Lrp3	low density lipoprotein receptor-related protein 3	5.465865	44.196651
Clstn2	calsyntenin 2	5.41628	42.703438
Adam5	a disintegrin and metallopeptidase domain 5	5.36495	41.210775
H2-M11	histocompatibility 2, M region locus 11	5.335531	40.378937
Ptx3	pentraxin related gene	5.320565	39.962237
Rtl3	retrotransposon Gag like 3	5.252916	38.131634
Gm26682	predicted gene, 26682	5.252803	38.128648
Morc1	microrchidia 1	5.251594	38.096689
Hoxc6	homeobox C6	5.176329	36.160165
Mcpt8	mast cell protease 8	5.146576	35.422062
Gabrb3	gamma-aminobutyric acid (GABA) A receptor, subunit beta 3	5.110873	34.556218
Sbspon	somatomedin B and thrombospondin, type 1 domain containing	5.085426	33.952042
Akap6	A kinase (PRKA) anchor protein 6	5.073102	33.663235
Hmcn1	hemicentin 1	5.060346	33.366905
Aqp2	aquaporin 2	5.031264	32.701036
Gria3	glutamate receptor, ionotropic, AMPA3 (alpha 3)	5.011924	32.265589
AI464131	expressed sequence AI464131	5.001414	32.03138
Muc13	mucin 13, epithelial transmembrane	4.997132	31.936452
Syt4	synaptotagmin IV	4.994666	31.881898
Spon1	spondin 1, (f-spondin) extracellular matrix protein	4.916033	30.190712
Caln1	calneuron 1	4.85996	29.039801
Hoxc4	homeobox C4	4.844155	28.723415
Tbx2	T-box 2	4.842042	28.681376
Jph2	junctophilin 2	4.816833	28.18456
Frzb	frizzled-related protein	4.79214	27.706253
Galnt5	polypeptide N-acetylgalactosaminyltransferase 5	4.77782	27.432606
Gm29100	predicted gene 29100	4.77255	27.332593
Tspyl5	testis-specific protein, Y-encoded-like 5	4.771509	27.312867
Spag17	sperm associated antigen 17	4.770653	27.296671
Ldoc1	regulator of NFKB signaling	4.7479	26.86954
4833422C13Rik	RIKEN cDNA 4833422C13 gene	4.730678	26.550693

Conversely, both CD45+ subsets were significantly up-regulated for GO pathways related to regulation of lymphocyte activation and proliferation, and innate immune cell responses (Figure 5D). Interestingly, among the top genes upregulated in the TdT^OSX^+ CD45+ subsets versus the CD45 single positive populations (Table 2), we found many genes expressed by tumor promoting immune cells, such as regulatory T cells (i.e. *Foxp3*) and gamma-delta T cells (i.e. *Tcrg-C1*). The most upregulated gene in the TdT^OSX^+;CD45+ subset (>80 fold versus CD45 single positive) was *Cd163l1*, a marker of gamma-delta IL-17 producing cells (Tan et al., 2019) and M2 macrophages (González-Domínguez et al., 2015), two tumor-promoting immune populations. We confirmed *Cd163l1* higher expression by RT-PCR together with other two genes, *Wdr78* and *Spock2*, also upregulated in the double positive cells (Figure 5—figure supplement 1). The second most expressed gene was Lymphotoxin beta (*Ltb*), a cytokine produced by lymphocytes and NK cells and associated with carcinogenesis (Wolf et al., 2010). The third one, *Klrg1*, negatively regulates cytotoxic lymphocytes and is associated with lymphocyte senescence and dysfunction (Henson and Akbar, 2009). Thus, these analyses revealed that two main transcriptional programs predominate based on CD45 expression, and suggest that TdT^OSX^+;CD45- comprise a subset of CAFs with some characteristics of skeletal cells, while TdT^OSX^+;CD45+ cells represent a heterogeneous subset of tumor-promoting immune cells.

**Table 2.** Top 50 uniquely up-regulated genes in tumor-derived TdT^OSX^+;CD45+ cells. List of the 50 most highly expressed genes in the TdT^OSX^+;CD45+ cells compared to the TdT^OSX^-;CD45+ cells isolated from the TME.

external_gene_ name	description	TdTOSX+CD45+ logFC	TdTOSX+CD45+ linearFC
Cd163l1	CD163 molecule-like 1	6.482112	89.394369
Ltb	lymphotoxin B	6.217037	74.389998
Klrg1	killer cell lectin-like receptor subfamily G, member 1	5.915697	60.367363
Foxp3	forkhead box P3	5.882423	58.991007
Trbv19	T cell receptor beta, variable 19	5.83901	57.242322
Cd5	CD5 antigen	5.822965	56.609196
Gm4759	predicted gene 4759	5.712238	52.426999
Tcrg-C1	T cell receptor gamma, constant 1	5.709921	52.342868
Icos	inducible T cell co-stimulator	5.588856	48.129715
Gm19585	predicted gene, 19585	5.577582	47.755056
Fasl	Fas ligand (TNF superfamily, member 6)	5.571314	47.548033
Rln3	relaxin 3	5.518062	45.824955
Ubash3a	ubiquitin associated and SH3 domain containing, A	5.432308	43.180511
Pcsk1	proprotein convertase subtilisin/kexin type 1	5.376329	41.537117
Ccr8	chemokine (C-C motif) receptor 8	5.32423	40.06386
Gimap3	GTPase, IMAP family member 3	5.257508	38.253185
Slamf1	signaling lymphocytic activation molecule family member 1	5.220641	37.288037
Klrb1f	killer cell lectin-like receptor subfamily B member 1F	5.204067	36.86212
Ikzf3	IKAROS family zinc finger 3	5.185332	36.386518
Trdv4	T cell receptor delta variable	5.179288	36.234394
Trat1	T cell receptor associated transmembrane adaptor 1	5.144648	35.374755
Pdcd1	programmed cell death 1	5.092017	34.107504
Izumo1r	IZUMO1 receptor, JUNO	5.053635	33.212045
Cd3e	CD3 antigen, epsilon polypeptide	5.042174	32.949246
Itk	IL2 inducible T cell kinase	5.027182	32.608632
Ctla4	cytotoxic T-lymphocyte-associated protein 4	4.978043	31.516668
Actn2	actinin alpha 2	4.919967	30.27316
Trbv1	T cell receptor beta, variable 1	4.891164	29.674756
Dkkl1	dickkopf-like 1	4.820628	28.258804
Cd300e	CD300E molecule	4.801355	27.883791
Pglyrp2	peptidoglycan recognition protein 2	4.80032	27.863797
Cd226	CD226 antigen	4.766441	27.21709
Themis	thymocyte selection associated	4.760082	27.097397
Dpep3	dipeptidase 3	4.694473	25.892693
Cd209a	CD209a antigen	4.650261	25.111233
Cd27	CD27 antigen	4.648725	25.084512
Gpr55	G protein-coupled receptor 55	4.621113	24.608975
Ccr6	chemokine (C-C motif) receptor 6	4.596004	24.18439
Gpr174	G protein-coupled receptor 174	4.594683	24.16225
A630023P12Rik	RIKEN cDNA A630023P12 gene	4.586499	24.025577
Cd28	CD28 antigen	4.564255	23.657981
Cd3g	CD3 antigen, gamma polypeptide	4.547609	23.386577
Lta	lymphotoxin A	4.534293	23.171712
Ccr3	chemokine (C-C motif) receptor 3	4.526527	23.047318
Gzmb	granzyme B	4.518658	22.921957
Lrrc66	leucine rich repeat containing 66	4.47147	22.184347
Xirp2	xin actin-binding repeat containing 2	4.462937	22.053513
St8sia1	ST8 alpha-N-acetyl-neuraminide alpha-2,8-sialyltransferase 1	4.460924	22.022769
Sit1	suppression inducing transmembrane adaptor 1	4.417363	21.367754
Tnfrsf4	tumor necrosis factor receptor superfamily, member 4	4.381868	20.84844

### Double positive TdT^OSX^+;CD45+ cells are a heterogeneous immune population enriched in lymphoid cells

**Figure 6.**
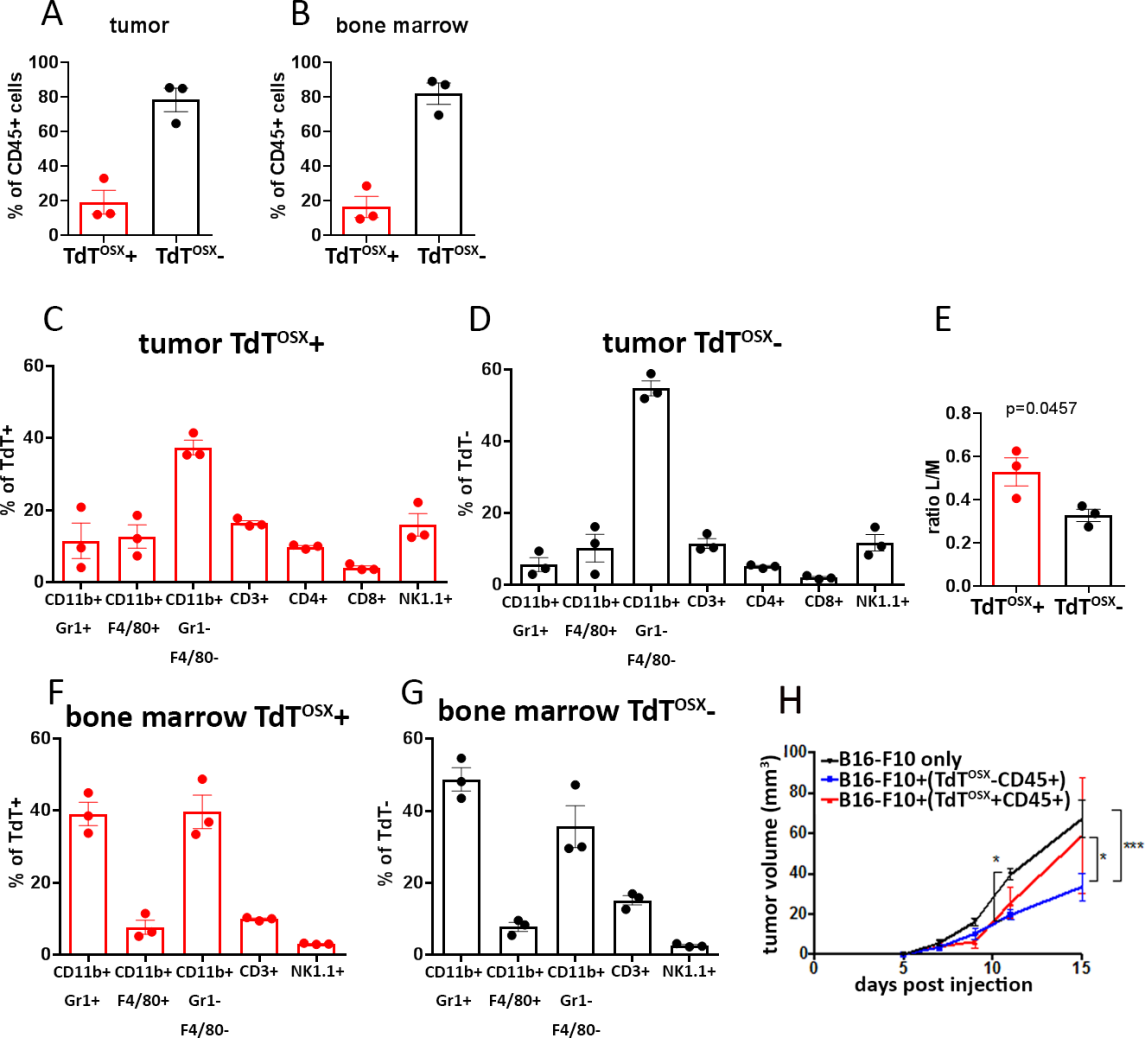
Subsets of myeloid and lymphoid cells in the tumor microenvironment and the bone marrow are derived from TdT^OSX^+ cells. (**A–B**) Quantification of FACS analysis showing the percentage of TdT^OSX^+;CD45+ and TdT^OSX^-;CD45+ populations in the tumor and bone marrow of Osx-cre;TdT mice injected subcutaneously with B16-F10 tumor cells. Data showed as % of total CD45+ cells. (**C–D**) Quantification of FACS analysis showing the percentage of tumor infiltrating myeloid and lymphoid populations within the TdT^OSX^+;CD45+ or TdT^OSX^-;CD45+ subsets, each considered as 100%. (**E**) Lymphoid over myeloid ratio within the tumor infiltrating TdT^OSX^+;CD45+ or the TdT^OSX^-;CD45+ subsets. Statistical analysis was performed by student t-test. (**F–G**) Quantification of FACS analysis showing the percentage of the bone marrow resident myeloid and lymphoid populations within the TdT^OSX^+;CD45+ or TdT^OSX^-;CD45+ subsets, each considered as 100%. n = 3/group. (**H**) Tumor growth in mice injected with B16-F10 tumor cells alone or together with tumor-derived TdT^OSX^+;CD45+ or TdT^OSX^-;CD45+ cells at the ratio 1:5. n = 3–6/group. Figure 6—source data 1.Relates to FACS analysis.

Next we performed flow cytometry analysis to better characterize the TdT^OSX^+;CD45+ population and validate some of the RNAseq findings using the B16-F10 melanoma model. We confirmed that the double positive TdT^OSX^+;CD45+ represented about 20% of the total CD45+ cells in both the primary tumor and the bone marrow of doxy-fed Osx-cre;TdT mice (Figure 6A and B). Next, we analyzed the percentage of TdT^OSX^+ expressing the common myeloid and lymphoid markers (gate strategy in Figure 6—figure supplement 1), such as CD11b (monocytes), F4/80 (macrophages), Gr1 (granulocytes/neutrophils), CD3 (T cells, further divided into CD4+ and CD8+), and NK1.1 (Natural Killer cells). To standardize the comparison between the TdT^OSX^+;CD45+ and TdT^OSX^-;CD45+ (single positive) populations within each sample, data were represented as percentage of either total TdT^OSX^+;CD45+ or total CD45 single positive cells (Figure 6C and D). TdT^OSX^+;CD45+ subset expressed both myeloid and lymphoid markers, with a similar pattern as the CD45 single positive cells. In both subsets, the majority of the tumor immune infiltrate included myeloid populations, which represented about 60% of the TdT^OSX^+;CD45+ and over 70% of the total CD45 single positive cells. Lymphoid cells appeared to be more abundant among the TdT^OSX^+;CD45+ (32.3 ± 6.5%), compared to the TdT^OSX^-;CD45+ cells (23.3 ± 4.3%). Based on this observation, we calculated the ratio of lymphoid over myeloid populations and observed that the tumor-infiltrating TdT^OSX^+;CD45+ double positive population was enriched in lymphoid cells (Figure 6E). Flow cytometric analysis of the bone marrow from tumor bearing Osx-cre;TdT mice revealed similar pattern of distribution between the TdT^OSX^+;CD45+ and the CD45 single positive immune populations (Figure 6F and G), although the frequencies of each immune subset in the bone marrow differed compared to the cells at tumor site. These data suggest that Osx marks multiple immune cell types but that TdT^OSX^+-derived immune cells infiltrating a tumor are skewed towards lymphoid populations.

**Figure 6—figure supplement 1.**
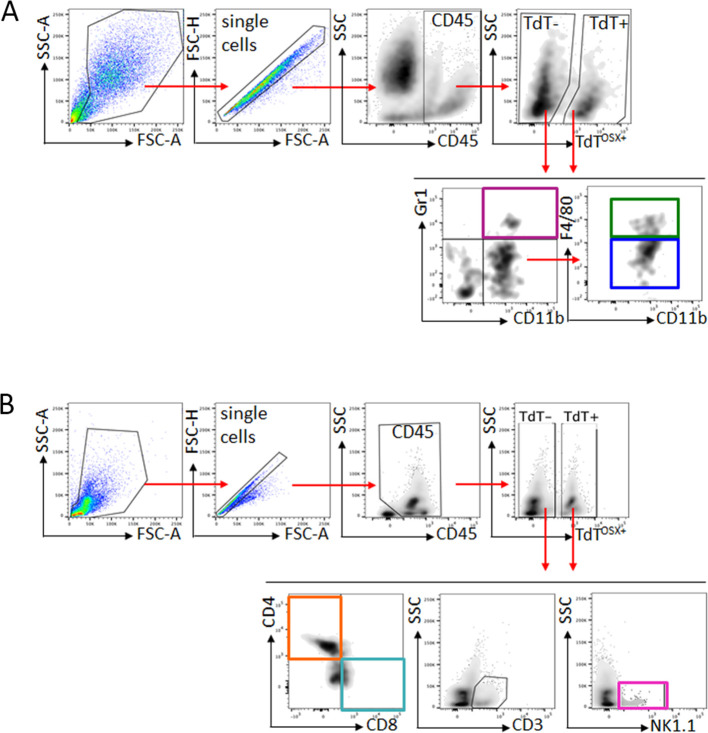
Gate strategy for immune staining of tumor and bone marrow. (**A**) Representative gate strategy analysis of total tumor mass stained for CD45, myeloid immune markers CD11b, F4/80 and Gr1. Analysis performed in both TdT^OSX^+ and TdT^OSX^- populations. Identical analysis was performed on BM samples. (**B**) Representative gate strategy analysis of BM stained for lymphoid immune markers CD3, CD8, CD4 and NK1.1. Analysis performed in both TdT^OSX^+ and TdT^OSX^- populations. Identical analysis was performed on tumor samples. Fluorescence minus one (FMO) controls were used to set the gates. WT;TdT mice were used to set the gate for TdT+ cells.

Since adoptive transfer of tumor-derived TdT^OSX^+ cells increases tumor growth (Figure 3B), we asked whether TdT^OSX^+;CD45+ immune populations contribute to tumor progression. We isolated by FACS sorting TdT^OSX^+;CD45+ and the TdT^OSX^-;CD45+ from B16-F10 subcutaneous tumors from doxy-fed Osx-Cre;TdT mice. Sorted cells were then re-injected along with B16-F10 tumor cells (ratio 5:1) into new WT recipient mice. The co-injection of CD45 single positive cells decreased tumor growth relative to B16-F10 injected alone at day 15, while TdT^OSX^+;CD45+ did not show anti-tumor effects (Figure 6H).

Collectively, these data indicate that *Sp7* marks cells of both mesenchymal and hematopoietic origin to support the development of tumor promoting populations.

### Double positive TdT^OSX^+;CD45+ cells derive from TdT^OSX^+ HSCs

**Figure 7.**
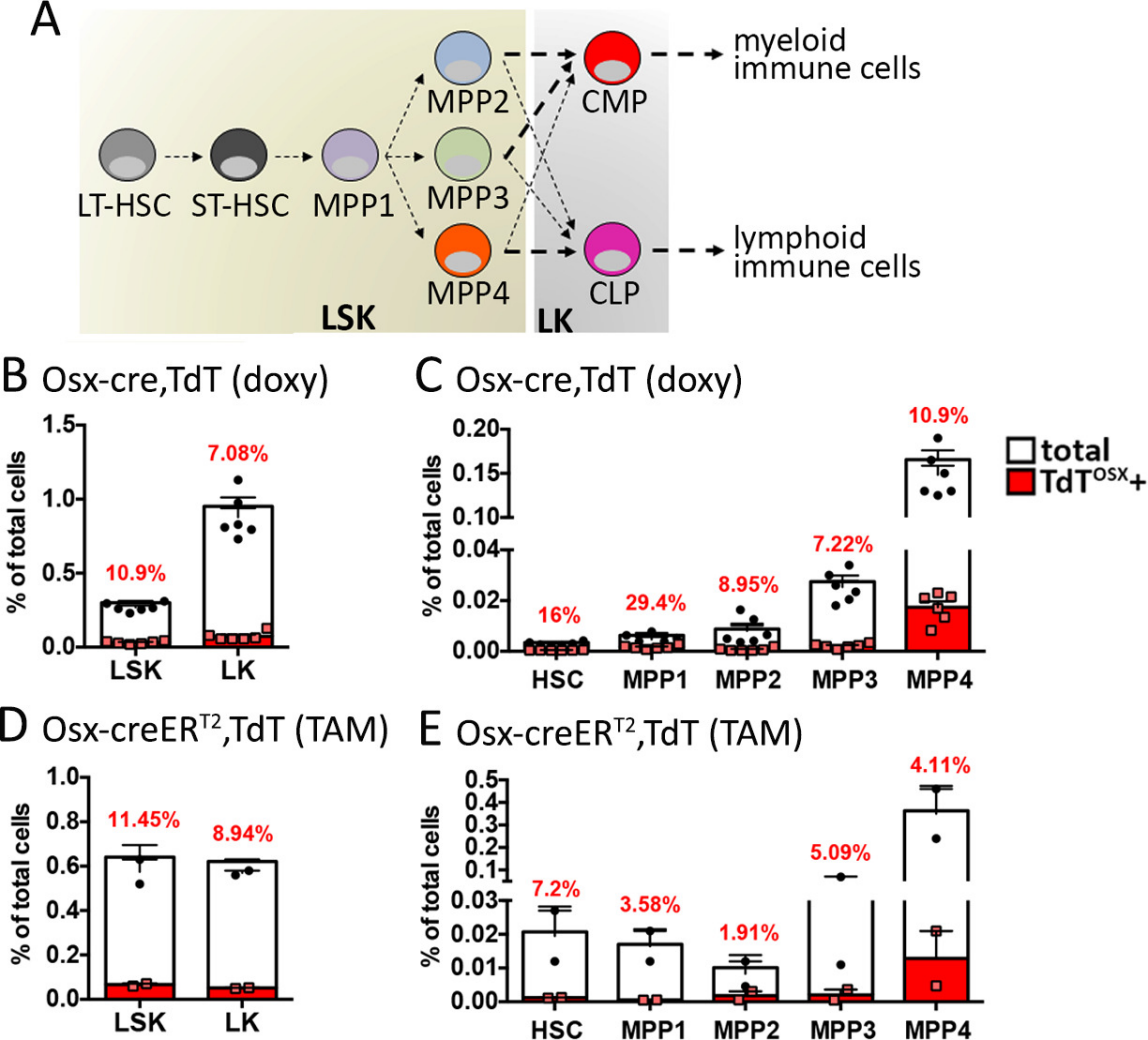
TdT^OSX^+;CD45+ cells derive from TdT^OSX^+ HSCs. (**A**) Schematic of hematopoietic differentiation. (**B–E**) Flow cytometry quantification of TdT^OSX^+ cells in the bone marrow of (**B–C**) doxy-fed Osx-cre;TdT mice and (**D–E**) TAM-treated Osx-creER^T2^;TdT mice injected subcutaneously at 7 weeks of age with B16-F10 tumors. In (**B and D**) LSK and LK subsets are shown, while in (**C and E**) the LSK population is further divided into HSCs and MPP1-4 subsets. Red numbers represent the average of TdT^OSX^+ cells in each specific subset. n = 2–6/group. Figure 7—source data 1.Relates to FACS analysis.

To determine whether *Sp7* may be activated at early stages of hematopoiesis as a potential explanation for the heterogeneity of TdT^OSX^+;CD45+ cells, we performed flow cytometric analysis for hematopoietic stem cell (HSC) markers in the bone marrow of doxy-fed Osx-cre;TdT and TAM-induced Osx-creER^T2^;TdT mice bearing B16-F10 tumors subcutaneously. We analyzed the lineage negative (Lin^-^) LSK and LK populations (Figure 7A) gated based on the expression of Sca1 and c-kit and found 10.9–11.45% TdT^OSX^+ LSK and 7.08–8.94% TdT^OSX^+ LK in Osx-cre;TdT and Osx-creER^T2^;TdT mice, respectively (Figure 7B and D). The LSK population was further divided into HSC and multipotent progenitor MPP(1-4) subsets (Figure 7A and gate strategy in Figure 7—figure supplement 1). Interestingly, TdT^OSX^+ cells represented about 30% of MPP1, and between 7–11% of the MPP2-MPP3 and MPP4 subsets in Osx-cre;TdT mice (Figure 7C). Similar results were also obtained in Osx-creER^T2^;TdT mice (Figure 7E). The slight difference in frequency between the two mouse models could depend on the different timing of Cre activation, but both models succeeded to find TdT^OSX^+ cells in HSCs.

**Figure 7—figure supplement 1.**
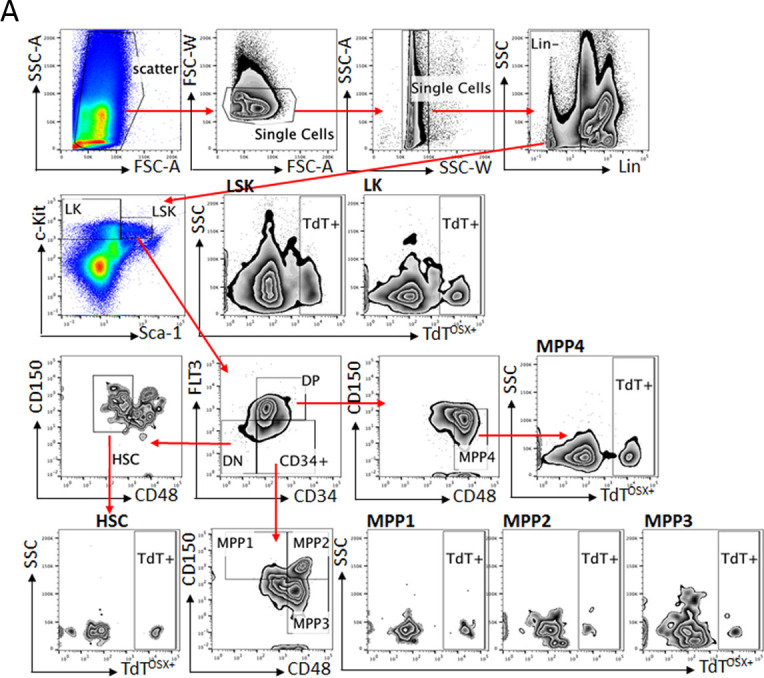
Gate strategy for HSC immunostaining of bone marrow. (**A**) Representative gate strategy analysis of bone marrow stained for HSC subsets. TdT^OSX^+ cells were quantified in each subset. Fluorescence minus one (FMO) controls were used to set the gates. WT;TdT mice were used to set the gate for TdT+ cells.

**Figure 8.**
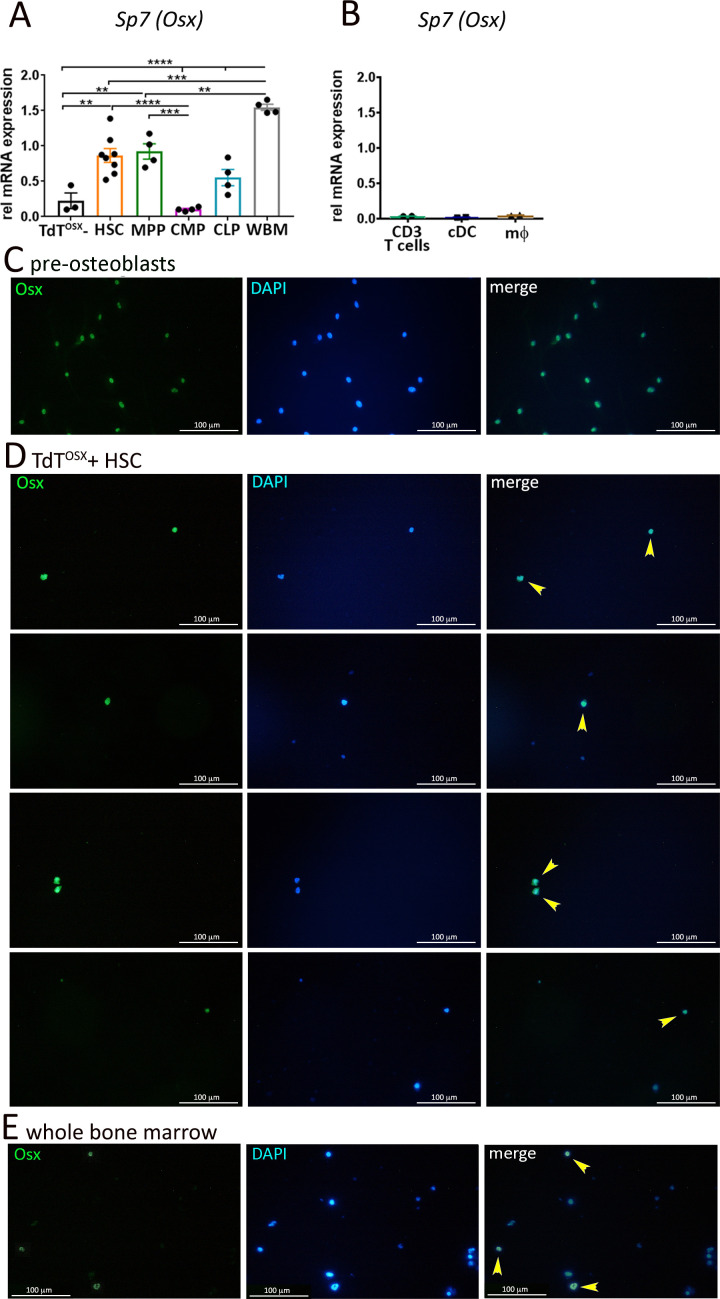
*Sp7* transcripts and Osx protein are expressed in a subset of HSCs isolated from bone marrow of naïve mice. (**A**) Real Time PCR analysis comparing sorted hematopoietic stem cells (HSC), multipotent progenitors (MPP), common myeloid progenitors (CMP), common lymphoid progenitors (CLP) and whole bone marrow (WBM) from 7 to 9 week old mice. FACS sorted TdT^OSX^- cells from the tumor of Osx-cre;TdT mice were used as negative control (n = 3–8/group). Statistical analysis was determined by two-way ANOVA followed by Tukey post-hoc test. *p<0.05, **p<0.01, ***p<0.001, ****p<0.0001. (**B**) Real Time PCR analysis of isolated mature CD3+ T cells, conventional dendritic cells (cDC) and bone marrow-derived macrophages (mφ) from WT naïve mice. (**C–E**) Immunofluorescence for Osx in (**C**) primary osteoblasts differentiated from BMSC cultured for 4 days in osteogenic media, (**D**) TdT^OSX^+ HSCs sorted from Osx-cre;TdT reporter mice and (**E**) whole bone marrow cells from WT mice. DAPI is used for nuclear staining. Magnification 200X. Figure 8—source data 1.Relates to Real-Time PCR in panels A, B.

Importantly, RT-PCR of isolated HSCs from the bone marrow of 7–8 week old wild-type naïve mice showed *Sp7* expression compared to the TdT^OSX^- fraction isolated from the tumor mass, used as negative control (Figure 8A). Similarly, *Sp7* transcripts were found in the MPP1-4 subsets, and in the common lymphoid progenitors CLP, but not in the common myeloid progenitors CMP (Figure 8A). In contrast, RT-PCR analysis of mature immune populations from naïve mice (macrophages, dendritic cells and T cells) failed to detect *Sp7* expression (Figure 8B). Finally, we performed immunostaining for Osx to confirm protein expression on a single cell level. To enrich for Osx+ cells, we FACS sorted TdT^OSX^+ HSCs from the bone marrow of Osx-cre;TdT reporter mice after enrichment for c-kit+ cells. Sorted cells were plated on serum-coated coverslips for two days before immunostaining for Osx and with DAPI for nuclear localization. We confirmed Osx localization in the nuclei of the majority of TdT^OSX^+ HSCs (Figure 8D). As positive control for Osx staining, we used bone marrow stromal cells isolated from WT mice cultured for 4 days in osteogenic medium (Figure 8C). Whole bone marrow was used as additional control confirming that only a small number of cells expressed Osx while the majority was negative (Figure 8E). Thus, for the first time we show that subsets of HSCs express the transcription factor *Sp7*, giving rise to tumor infiltrating immune populations.

## Discussion

Numerous studies have established the importance of the TME for primary tumor growth and metastatic dissemination (Goubran et al., 2014). In skeletal metastases, crosstalk between bone residing cells, osteoblasts and osteoclasts, and tumor cells drives tumor growth (Weilbaecher et al., 2011). Recent studies also suggest that osteoblasts and their secretory products stimulate the expansion of bone marrow-derived myeloid populations, which in turn escape the bone marrow and reach distant sites to support tumor growth by inhibiting anti-tumor immune responses (D'Amico et al., 2016; Engblom et al., 2017). Our study demonstrates that a transcription factor required for osteoblast differentiation, Osterix, marks pro-tumorigenic stromal cells infiltrating extra-skeletal tumors. A possible role of Osx in tumorigenesis is emerging from human studies as well showing that presence of *Sp7* in the tumor is associated with poor patient survival (Yao et al., 2019). Our own analysis of published gene microarray data of breast cancer stroma (Finak et al., 2008), reveals higher *Sp7* expression in tumor stroma relative to healthy tissue from the same subject, corroborating the notion that Osx+ stromal cells regulate tumor growth. Therefore, Osx function may well extend beyond its known role in bone development and homeostasis.

Osterix has been primarily studied in the context of osteoblast differentiation and regulation of bone mass. *Sp7* deficient mice die within 1 hr of birth with a complete absence of intramembranous and endochondral bone formation (Nakashima et al., 2002; Baek et al., 2009). During embryogenesis and perinatally, *Sp7* is expressed in MSCs in the bone marrow, and it is necessary for the full osteogenic program. In adult mice, *Sp7* is mainly confined to committed osteoblasts and osteocytes. There is increasing evidence of *Sp7* expression in cells residing outside the skeleton, including synovial fibroblasts (Miura et al., 2019), dental pulp (Monterubbianesi et al., 2019), olfactory glomerular cells, gastric and intestinal epithelium (Chen et al., 2014), and kidney (Strecker et al., 2013). Here we demonstrate that *Sp7* is also present in a CAF subset, and in hematopoietic precursors, thus challenging the dogma that *Sp7* is exclusively expressed by mesenchymal cells. Our cell tracking studies using the repressible Tet-OFF Osx-cre;TdT reporter and the tamoxifen-inducible Osx-creER^T2^;TdT model demonstrate the presence of TdT^OSX^+;CD45+ cells in the bone marrow of adult mice. At least two other independent studies have also shown abundant and persistent Osx+ cells in the bone marrow when the reporter gene is activated either embryonically or neonatally (Liu et al., 2013; Mizoguchi et al., 2014), and at 3 weeks of age (Strecker et al., 2013), but no signal in the bone marrow when the Osx-cre is activated after 8 weeks of age (Mizoguchi et al., 2014). Notably, most of the studies with Osx-cre reporter mice have excluded the hematopoietic marker CD45 in the analysis of the Osx+ populations, and no report to date has shown *Sp7* expression in the hematopoietic compartment. Importantly, we also report that bone marrow residing TdT^OSX^+ populations expand during tumor progression. Such result is highly unlikely linked to systemic factors released from extra-skeletal tumors enhancing the Tet-transactivator in the Tet-OFF;Osx-cre;TdT mice. In fact, we observed increased TdT^OSX^+ cells (CD45- and CD45+ subsets) also in the bone marrow of tumor bearing Osx-creER^T2^;TdT animals. Our findings are instead consistent with previous observations reporting increased numbers of bone marrow residing MSCs and osteoblast precursors in mice and patients with lung adenocarcinomas (Engblom et al., 2017), and with the reprogramming of hematopoiesis towards increased myelopoiesis during tumor progression (Capietto et al., 2013; Meyer et al., 2018).

Here, we report for the first time that a subset of hematopoietic lineage cells in the bone marrow and at tumor site derives from an Osx+ progenitor, which is present embryonically and persists until 7–8 weeks of age. Importantly, we detected *Sp7* transcripts and Osx nuclear localization in a subset of HSCs. These results support the observation that TdT^OSX^+;CD45+ cells are very heterogeneous and express monocyte, macrophage, granulocyte, T cell and NK cell markers. The heterogeneity of the TdT^OSX^+;CD45+ populations is most likely derived from expression of *Sp7* in HSC and MPP subsets. These precursors can give rise to the lineage committed common lymphoid (CLP) and myeloid (CMP) progenitors that differentiate into all the mature immune cells (Pietras et al., 2015). *Sp7* transcripts are also detected in CLP but not in CMP or mature immune populations, indicating that Osx is activated early during hematopoiesis, with higher expression in cells committed to the lymphoid lineage. Despite expressing both myeloid and lymphoid markers, we noted increased in lymphoid over myeloid ratio in the TdT^OSX^+;CD45+ tumor infiltrating cells relative to the TdT^OSX^-;CD45+ cells. TdT^OSX^+;CD45+ subset is also enriched in genes expressed by immunosuppressive or exhausted lymphocytes. Such result is consistent with the functional tumor co-injection studies showing that TdT^OSX^-;CD45+ cells reduce tumor growth while the TdT^OSX^+;CD45+ subset does not.

Co-injection of tumor cells with cells sorted from the primary tumor has been used to validate the pro-tumorigenic roles of CAFs and certain immune suppressive CD45+ populations. Here, we show that tumor-derived TdT^OSX^+ cells, comprising both CD45 positive and negative subsets, increase tumor growth compared to tumor cells injected alone. By contrast, co-injection of bone marrow-derived TdT^OSX^+ cells with the tumor cells does not exert any pro-tumor effect suggesting that bone marrow-derived TdT^OSX^+ cells are functionally distinct from tumor-derived TdT^OSX^+ cells. Hence, if tumor-derived TdT^OSX^+ cells originate from the bone marrow in response to an incipient tumor, as it would be anticipated by their increase in the circulation of tumor-bearing mice, they must undergo a conditioning process within the TME to acquire a pro-tumorigenic function. Because Osx regulates expression of proteins required for the generation of the bone tissue, it is likely that a main role of Osx+ mesenchymal cells (TdT^OSX^+;CD45-) in the TME is to produce and remodel extracellular matrix. Fibroblasts with an osteoblast signature involved in matrix production have been recently identified by single cell RNA sequencing in rheumatoid arthritis (Croft et al., 2019). ECM accumulation is quite frequent within the TME, causing in more severe cases an intense fibrotic response, or desmoplasia, and tumor stiffening (Gkretsi and Stylianopoulos, 2018). Stiffening is not only required for a primary tumor to displace the host tissue and grow in size, but also contributes to cell–ECM interactions and can promote cancer cell invasion to surrounding tissues (Gkretsi and Stylianopoulos, 2018). As noted earlier, upregulation of *Sp7* in breast cancer cells is associated with increased invasion and bone metastasis, and this may occur by upregulation of ECM modifying metalloproteinases, MMP9 and MMP13, and other factors that increase vascularization, and affect bone cell function (Yao et al., 2019).

Since *Sp7* is not expressed in mature immune populations, it is difficult to envision a role for Osx in modulating anti-tumor immune responses. Co-injection experiments indicate that TdT^OSX^+;CD45+ cells have better pro-tumorigenic function than TdT^OSX^–;CD45+ cells. However, only the co-injection of the bulk TdT^OSX^+ cells increases tumor growth over the B16-F10 tumor cells alone, suggesting that the Osx+ mesenchymal population (TdT^OSX^+;CD45-) is responsible for enhancing tumor growth. This finding is intriguing since the TdT^OSX^+;CD45- mesenchymal cells represent only 5% of the total tumor infiltrating TdT^OSX^+ populations. One could speculate that TdT^OSX^+;CD45- cells represent a subset of highly pro-tumorigenic CAFs, which can support tumor progression even when present in very limited numbers. Another possibility is that the TdT^OSX^+;CD45- mesenchymal cells might be required to create and maintain an immune suppressive environment where the CD45+ populations fail to exert anti-tumor effects.

In conclusion, we demonstrate that *Sp7* is expressed in a subset of tumor infiltrating mesenchymal cells with CAF and osteogenic cell features. Surprisingly, *Sp7* expression is also found in hematopoietic precursors, and marks tumor infiltrating immune populations enriched in immune suppressive markers. Considering the emerging data that *Sp7* expression in the tumor cells is linked to tumor progression, our results emphasize the importance of Osx in the TME and the need to evaluate the prognostic value of stromal *Sp7* expression in the patients.

## Materials and methods

**Key resources table:** 

Reagent type (species) or resource	Designation	Source or reference	Identifiers	Additional information
Gene (*Mus musculus*)	Sp7(Osx)	NCBI Gene	RRID:MGI:2153568	NCBI ID:170574
Genetic reagent (*Mus musculus*)	Tg(Sp7-tTA,tetO-EGFP/cre)1Amc/J (Osx-cre)	The Jackson Laboratories	Cat#006361 RRID:MGI:3689350	C57Bl/6
Genetic reagent (*Mus musculus*)	B6.Cg-Gt(ROSA)26Sortm9(CAG-tdTomato)Hze/J (TdT)	The Jackson Laboratories	RRID:MGI3813511	C57Bl/6
Genetic reagent (*Mus musculus*)	Sp7-creERT2/Ai9tdTomato (Osx-creERT2;TdT)	PMID:31768488	Cat#007909 RRID:MGI:4829803	Prof. Silva MJ (Washington University in St Louis) C57Bl/6
Cell line (*Mus musculus*, C57Bl/6)	B16-F10 melanoma cell line	ATCC	RRID:CRL-6475-LUC2	C57Bl/6
Cell line (*Mus musculus*)	PyMT breast cancer cell line	PMID:27216180		Prof. Weilbaecher KN (Washington University in St Louis) C57Bl/6
Cell line (*Mus musculus*)	Immortalized Cancer Associated Fibroblast cell line	PMID:27264173		Prof Longmore GD (Washington University in St Louis)
Antibody	Rat anti-mouse CD45-APCeFluor780(clone 30-F11)	eBioscience	Cat #47-0451-82 RRID:AB_1548781	FACS (1:400)
Antibody	Rat anti-mouse CD45-PE Cy7 (clone 30-F11)	eBioscience	Cat #25-0451-82 RRID:AB_2734986	FACS (1:400)
Antibody	Rat anti-mouse CD11b-AlexaFluor700 (clone M1/70)	eBioscience	Cat#56-0112-82 RRID:AB_657585	FACS (1:400)
Antibody	Rat anti-mouse Gr1(Ly6G)-FITC (clone RB6-8C5)	Miltenyi Biotec	Cat#130-102-837 RRID:AB_2659858	FACS (1:100)
Antibody	Rat anti-mouse F4/80 PerCP Cy5.5 (clone BM8)	Biolegend	Cat#123126 RRID:AB_10802654	FACS (1:400)
Antibody	Hamster anti-mouse CD3e-PE (clone 145–2 C11)	eBioscience	Cat#A14714 RRID:AB_2534230	FACS (1:200)
Antibody	Rat anti-mouse CD4-APC (clone RM4-5)	BD Biosciences	Cat#553051 RRID:AB_398528	FACS (1:200)
Antibody	Rat anti-mouse CD8a-FITC (clone 53–6.7)	BD Biosciences	Cat#561966 RRID:AB_10896291	FACS (1:200)
Antibody	Mouse anti-mouse NK1.1 (clone PK136)	Biolegend	Cat#108722 RRID:AB_2132712	FACS (1:200)
Antibody	Rat anti-mouse CD45R (B220)-Biotin (clone RA3-6B2)	eBioscience	Cat#13-0452-82 RRID:AB_466449	FACS (0.4 ul/sample)
Antibody	Hamster anti-mouse CD3e-Biotin (clone 145–2 C11)	eBioscience	Cat#13-0031-82 RRID:AB_466319	FACS (0.4 ul/sample)
Antibody	Rat anti-mouse Ter119 (clone TER119)	eBioscience	Cat# 13-5921-82 RRID:AB_466797	FACS (0.4 ul/sample)
Antibody	Rat anti-mouse Ly-6G/Ly-6C Biotin (clone RB6-8C5),	eBioscience	Cat#13-5931-82 RRID:AB_466800	FACS (0.2 ul/sample)
Antibody	Rat anti-mouse CD41Biotin (clone MWReg30)	eBioscience	Cat#13-0411-82 RRID:AB_763484	FACS (1 ul/sample)
Antibody	Streptavidin-BV510	BD Biosciences	Cat#563261	FACS (0.5 ul/sample)
Antibody	Rat anti-mouse Sca-1 BV711 (clone D7)	BD Biosciences	Cat#563992 RRID:AB_2738529	FACS (0.25 ul/sample)
Antibody	Rat anti-mouse c-kit(CD117)-APCeFluor780 (clone ACK2)	eBioscience	Cat#47-1172-82 RRID:AB_1582226	FACS (1 ul/sample)
Antibody	Rat anti-mouse CD16/32-BUV395 (clone 2.4G2)	BD Biosciences	Cat#740217 RRID:AB_2739965	FACS (0.5 ul/sample)
Antibody	Rat anti-mouse CD34-FITC (clone RAM34)	eBioscience	Cat#11-0341-82 RRID:AB_465021	FACS (4 ul/sample)
Antibody	Rat anti-mouse CD150(SLAM)-BV421 (clone TC15-12F12.2)	Biolegend	Cat#115943 RRID:AB_2650881	FACS (1 ul/sample)
Antibody	Hamster anti-mouse CD48-PE Cy7 (clone HM48-1)	eBioscience	Cat#25-0481-80 RRID:AB_1724087	FACS (0.15 ul/sample)
Antibody	Rat anti-mouse CD135(Flt3)-APC (clone A2F10)	eBioscience	Cat#17-1351-82 RRID:AB_10717261	FACS (2 ul/sample)
Antibody	Rat anti-mouse CD45 (clone 30-F11)	Invitrogen	Cat#14-0451-82 RRID:AB_467251	IF, IHC (1:200)
Antibody	Rabbit anti-mouse RFP/TdT	Rockland	Cat#600-401-379 RRID:AB_2209751	IF,IHC (1:500)
Antibody	Goat-anti-Rat IgG-Biotin	Thermo Fisher	Cat#31830 RRID:AB_228355	IF, IHC (1:500)
Antibody	Streptavidin-HRP	Bio-Rad	Cat#STAR5B	IF, IHC (1:500)
Antibody	Goat-anti-rabbit Alexa-fluor647	Thermo Fisher	Cat#A21244 RRID:AB_2535812	IF (4 ug/ml)
Antibody	Rabbit anti-mouse Sp7/Osx	Abcam	Cat#ab227820	IF (1:500)
Antibody	Goat anti-rabbit AlexaFluor488	Abcam	Cat#ab150077 RRID:AB_2630356	IF (1:1000)
Sequence-based reagent	Cyclophilin	This paper	PCR primers	5’-AGC ATA CAG GTC CTG GCA TC-3’ and 5’-TTC ACC TTC CCA AAG ACC AC-3’
Sequence-based reagent	Sp7(Osx)	This paper	PCR primers	5’-AAG GGT GGG TAG TCA TTT GCA-3’ and 5’-CCC TTC TCA AGC ACC AAT GG-3’
Sequence-based reagent	S100A4(Fsp-1)	This paper	PCR primers	5’-TGA GCA ACT TGG ACA GCA ACA-3’ and 5’-TTC CGG GGT TCC TTA TCT GGG-3’
Sequence-based reagent	Acta2(α-SMA)	This paper	PCR primers	5’-GTC CCA GAC ATC AGG GAG TAA-3’ and 5’-TCG GAT ACT TCA GCG TCA GGA-3’
Sequence-based reagent	Col1a1	This paper	PCR primers	5’-GGC CTT GGA GGA AAC TTT GC-3’ and 5’-GGG ACC CAT TGG ACC TGA AC-3’
Sequence-based reagent	Bglap(Ocn)	This paper	PCR primers	5’-GGA CTG AGG CTC TGT GAG GT-3’ and 5’-CAG ACA CCA TGA GGA CCA TC-3’
Sequence-based reagent	Runx2	This paper	PCR primers	5’-GTT ATG AAA AAC CAA GTA GCC AGG-3’ and 5’-GTA ATC TGA CTC TGT CCT TGT GGA-3’
Sequence-based reagent	BSP	This paper	PCR primers	5’-AGG ACT AGG GGT CAA ACA C-3’ and 5’-AGT AGC GTG GCC GGT ACT TA-3’
Sequence-based reagent	TNAP	This paper	PCR primers	5’-GGG GAC ATG CAG TAT GAG TT-3’ and 5’-GGC CTG GTA GTT GTT GTG AG-3’
Commercial assay or kit	TSA FITC System	Perkin Elmer	Cat#NEL701A001KT	
Commercial assay or kit	BOND Intense R detection kit	Leica Biosystem	Cat#DS9263	
Commercial assay or kit	BOND Polymer Refine Red detection kit	Leica Biosystem	Cat#DS9390	
Commercial assay or kit	Avidin/Biotin Blocking kit	Vector Labs	Cat#SP2001 RRID:AB_2336231	
Software, algorithm	STAR	STAR	RRID:SCR_015899	Version 2.0.4b
Software, algorithm	Subread	Subread	RRID:SCR_009803	Version 1.4.5
Software, algorithm	R	R	RRID:SCR_001905	Version 3.4.1
Software, algorithm	EdgeR	EdgeR	RRID:SCR_012802	Version 3.20.2
Software, algorithm	LIMMA	LIMMA	RRID:SCR_010943	Version 3.34.4
Software, algorithm	GAGE	GAGE	RRID:SCR_017067	Version 2.28.0
Other	VECTASHIELD Mounting Medium with DAPI	Vector Labs	Cat#H1200 RRID:AB_2336790	one drop
Other	ProLong Gold with DAPI	Invitrogen	Cat#P10144	one drop

### Tumor cell lines, CAF isolation and generation of primary cell cultures

B16-F10 (C57BL/6 mouse melanoma cell line, ATCC # CRL-6475-LUC2), PyMT and PyMT-BO1-GFP (Su et al., 2016) (C57BL/6 mouse breast cancer cell lines), kindly provided by Prof. Katherine N Weilbaecher (Washington University in St. Louis), were cultured at 37°C in complete media (DMEM supplemented with 2 mM l-glutamine, 100 μg/ml streptomycin, 100 IU/ml penicillin, and 1 mM sodium pyruvate) containing 10% FBS. Immortalized CAF cell line was kindly provided by Prof. Gregory D Longmore (Washington University in St. Louis). All the cell lines used have been tested for mycoplasma and were mycoplasma free. CAFs were isolated from primary tumors as described in Corsa et al., 2016. Tumors were minced, digested and plated as single cell suspension on a tissue plastic dish for 30 min to separate the adherent fraction, composed of CAFs, from the cells in suspension, comprising of tumor cells, tumor infiltrating immune populations and endothelial cells, among few others. CAFs were then left 24 hr in culture and RNA was extracted afterwards.

Bone marrow stromal cells (BMSCs) were cultured in complete alpha-MEM (without ascorbic acid) containing 10% FBS and differentiated for 4 days in osteogenic medium (complete alpha-MEM supplemented with 50 μg/ml ascorbic acid and 10 μM beta-glycerophosphate) to obtain pre-osteoblasts.

Sorted hematopoietic stem cells (HSCs) were plated overnight on a glass coverslip coated with serum in the presence of StemPRO (Gibco) media and then fixed and used for immunostaining.

### Mouse strains and tumor models

Animals were housed in a pathogen-free animal facility at Washington University (St. Louis, MO, USA). Age and sex-matched animals were used in all experiments according to IACUC guidelines. *B6.Cg-Tg(Sp7-tTA,tetO-EGFP/cre)1Amc/J* (catalog #006361; The Jackson laboratory, ME USA) mice, which carry a tetracycline-responsive Osx promoter driving Cre (Osx-cre) were mated with reporter mice *B6.Cg-Gt(ROSA)26Sor^Tm9(CAG-tdTomato)Hze^/J* (TdT) (catalog #007909; The Jackson Laboratory), to generate Osx-cre;TdT mice. To suppress Cre expression, 200ppm doxycyline (doxy) was added to the chow (Test Diet #1816332–203, Purina, MO USA) and fed to some groups of mice until weaning (P28); pups were switched to standard rodent chow at weaning. *Sp7-creER^T2^/Rosa26^<fs-TdTomato>^* mice were kindly provided by Prof. Matthew D Silva (Washington University in St. Louis). Tamoxifen (#T5648, Millipore Sigma, MO USA) was dissolved in corn oil and injected at 100 mg/kg intra-peritoneally. Wild-Type (WT) C57BL/6 mice were purchased from The Jackson Laboratory.

Subcutaneous (sq) injections were performed using 10^5^ B16-F10 tumor cells suspended in 100 µl of sterile PBS and Matrigel Matrix (Corning #354234), while 10^5^ PyMT were suspended in 50 µl of sterile PBS and Matrigel Matrix and injected into the MFP. Adoptive transfer and co-injection with tumor cells were performed using a 5:1 ratio of TdT^OSX+^:B16-F10 tumor cells. The number of cells injected in each experiment varied depending upon the number of cells obtained from sorting in each experiment (hence, the differences in tumor size among the different experiments), but within (2.5×10^5^:5×10^4^) and (5×10^5^:1×10^5^) cells. B16-F10 cells only were injected as control using the same number of tumor cells co-injected with TdT^OSX^+ cells. Tumors were monitored by caliper measurements and mice were sacrificed between day 14 and 16 post tumor inoculation. Tumor volume was calculated according to the formula: 0.5236*length*(width [Kalluri and Zeisberg, 2006]).

### Flow cytometry and sorting

Single cell suspensions were prepared from fresh bone marrow and tumors upon sacrifice. Bone marrow cells were obtained from femurs and tibias by centrifugation, while tumors were minced and digested with 3.0 mg/ml collagenase A (Roche, Basel Switzerland) and 50 U/ml DNase I (Millipore Sigma) in serum-free media for 45 min at 37°C. Cells were filtered through 70 μm strainers, red blood cells (RBC) were then removed with RBC lysis buffer (Millipore Sigma), washed twice in PBS and stained in FACS buffer (0.5% BSA, 2 mM EDTA, 0.01%NaN_3_) with the following antibodies: CD45 (clone 30-F11, eBioscience), CD11b (clone M1/70, eBioscience), Ly6G (Gr-1) (clone RB6-8C5, eBioscience), F4/80 (BM8, Biolegend), CD3 (145–2 C11, eBioscience), CD4 (RM4-5, BD Biosciences), CD8 (clone 53–6.7, BD Biosciences), NK1.1 (clone PK136, Biolegend). Once stained, cells were sorted for the adoptive transfer, RT-PCR and RNAseq studies or were fixed for flow cytometry (gate strategy in Figure 7—figure supplement 1) in BD Cytofix fixation buffer (BD Biosciences, 554655). Samples were acquired using the BD LSR-Fortessa cytometer and analyzed with FlowJo version 9.3.2.

Hematopoietic stem cell subsets were analyzed from fresh bone marrow isolated from femurs, tibias and hip bones crushed with mortar and pestle in 3 ml of PBS, filtered in a 70μm-nylon strainer to remove bone fragments and lysed of red blood cells (RBC). 10 million cells were stained for each sample using the following antibodies: CD3, Gr1, B220 (clone RA3-6B2, eBioscience), Ter119 (clone TER119, e Bioscience), CD41 (clone eBioMWReg30, eBioscience) for lineage negative gating, Sca-1 (clone D7, BD Biosciences), CD117/c-kit (clone ACK2, eBioscience), CD16/32 (clone 2.4G2, BD Biosciences), CD34 (clone RAM34, eBioscience), CD150/SLAM (clone TC15-12F12.2, Biolegend), CD48 (clone HM48-1, eBioscience), CD135/Flt3 (clone A2F10, eBioscience), gate strategy in Figure 7—figure supplement 1. Once stained, cells were fixed in BD Cytofix fixation buffer, acquired using the Bio-Rad ZE5 (YETI) cytometer and analyzed with FlowJo version 10.4.1.

Isolation of hematopoietic progenitor subsets for Real Time analysis was obtained by FACS sorting using the following markers: HSCs (Lineage- c-Kit+Sca-1+CD48-CD150+), MPPs (Lineage- c-Kit+Sca-1+CD48-CD150-), CLPs (Lineage- c-Kit+Sca-1+IL7rα+Flt3+Ly6D-) and CMPs (Lineage- c-Kit+Sca-1-CD34+CD16/32). For IF, TdTomato was also used to sort Osx+ HSCs.

Isolation of hematopoietic stem cells for Osx immunostaining was performed as following: whole bone marrow was isolated from femurs, tibias and iliac crests and incubated with mouse CD117-conjugated microbeads (Miltenyi Biotec #130-091-224). CD117-enrichment was then carried out using the AutoMACS Pro Seperator (Miltenyi Biotec). Post CD117 enrichment, the positive cell fraction was stained to sort phenotypically defined TdTomato+ HSCs (Lineage- c-Kit+Sca-1+CD48-CD150+) by FACS.

### Histology

Tumors and bones (femur and tibia) were fixed in 4% PFA over-night (ON) at 4°C. The fixed bones were partially decalcified in 14% EDTA for 3 days, washed in a sucrose gradient (1 hr in 10% sucrose, 1 hr in 20% sucrose, ON in 30% sucrose) before snap-freezing them in OCT embedding medium. Tumors were directly put in sucrose gradient and then embedded in OCT. Frozen sections were cut at 5 μm thickness and kept at −20°C until analysis.

Thawed sections were washed in PBS and mounted with VECTASHIELD Mounting Medium containing DAPI (Vector Laboratories, CA USA).

Images were taken using the Leica DMi8 Confocal Microscopy at the Musculoskeletal Research Center (Washington University in St Louis), magnification 200X.

### Immunohistochemical staining

Tissues were fixed in 10% neutral-buffered formalin for 18 hr, embedded in paraffin after graded-ethanol dehydration, and sectioned into 6 μm sections using a microtome. Automated staining was carried out on the BondRxm (Leica Biosystems). Following dewaxing and citrate-buffered antigen retrieval, sections were stained with the primary antibody for 1 hr at RT. Sequential chromogenic detection was performed with the Bond IntenseR (Rat primary) and Bond Polymer Refine Red (Rabbit primary) detection kits (Leica Biosystems). The primary antibodies used were rat anti-CD45 (Invitrogen clone 30-F11, #14-0451-82) followed by rabbit anti-RFP/TdT (Rockland, # 600-401-379). Stained sections were dehydrated in graduated ethanol and xylene washes then mounted with xylene-based Cytoseal (Thermo Fisher).

Stained slides were imaged at 20X magnification with the Zeiss Axioscan slide scanner. For image analysis, Halo v3 (Indica Labs) was used to deconvolve dual stained images into single channels and pseudo colored with blue representing nuclei (hematoxylin), green representing CD45+ cells (DAB stained), and red representing Osx+ cells (RFP stained).

### Immunohistochemestry and immunofluorescence staining

For colocalization of TdT+ cells and CD45 in B16-F10 soft tissue tumors, subcutaneous tumors were dissected and fixed in 10% neutral-buffered formalin for 18 hr, embedded in paraffin after graded-ethanol dehydration, and sectioned into 6 μm sections using a microtome. Sections were dewaxed in xylene and then hydrated through graded ethanol washes. Endogenous peroxidases were quenched by incubating in hydrogen peroxide (1% in PBS) for 10 min at RT. Antigen retrieval was then performed by microwave treatment in a citrate buffer for 15 min. Sections were washed in TBS-T (1X TBS with 0.05%Tween-20) followed by blocking for 30 min at RT in blocking buffer (5% goat serum, 2.5% BSA in TBS). Slides were then blocked using the Avidin/Biotin Blocking Kit (Vector Labs). For staining, slides were incubated overnight in a humidified chamber at 4°C with 1:200 rat anti-CD45 (Invitrogen clone 30-F11, #14-0451-82) and 1:400 rabbit anti-RFP (Rockland, #600-401-379) antibodies diluted in 50% blocking buffer. After primary staining, slides were washed in TBS-T and then incubated with HRP-conjugated anti-rat IgG secondary (1:500 in 50% blocking buffer) for 30 min at RT. For fluorescent detection of CD45, slides were washed and then incubated in 1:50 FITC Tyramide reagent (PerkinElmer, # NEL701A001KT) for 8 min at RT. For fluorescent detection of RFP, slides were washed and incubated in AlexaFluor 594-conjugated anti-rabbit IgG secondary (1:500 in 50% blocking buffer) for 30 min at RT. Stained slides were washed and mounted using ProLong Gold with DAPI (Invitrogen).

#### Osx staining

HSCs, BMSC or whole bone marrow cells were plated overnight on coverslips coated with FBS for 30 min at RT then cells. Cells were fixed in 4% PFA for 15 min, washed and permeabilized with PBS/0.3%TritonX-100 for 3 min. Blocking was performed with 5% normal serum in permeabilization buffer for 1 hr at RT. Anti-Osx antibody (Abcam, #ab227820) was dissolved 1:500 in a buffer containing PBS/0.3%TritonX-100/1%BSA and incubated ON at 4°C. For fluorescent detection, coverslips were washed and incubated in AlexaFluor 488-conjugated anti-rabbit secondary antibody (1:1000) in PBS/0.3%TritonX-100/1%BSA for 1 hr at RT. Stained slides were washed and mounted using VECTASHIELD Mounting Medium containing DAPI (Vector Laboratories, CA USA). Fluorescent signals were captured by using a Nikon Eclipse 80i microscope and a Nikon DS-Qi1MC camera (Nikon, CO, USA).

### Real Time PCR

Total RNA was extracted with TRIzol (Invitrogen, CA USA) and quantified on a ND-1000 spectrophotometer (NanoDrop Technologies). The cDNA was synthesized with 1 μg RNA using High Capacity cDNA Reverse Transcription Kit (#4368814, Applied Biosystems, CA USA).

For purified hematopoietic precursors (HSC, MPP, CLP and CMP) total RNA was extracted using the NucleoSpin RNA XS kit (Macherey-Nagel #740902) and reverse transcribed with the SuperScript VILO kit (Invitrogen #11754–050).

The amount of each gene was determined using Power SYBR Green mix on 7300 Real-Time PCR System (Applied Biosystems). Cyclophilin mRNA was used as housekeeping control. Specific primers for mice were as follows: *Cyclophilin*, 5’-AGC ATA CAG GTC CTG GCA TC-3’ and 5’-TTC ACC TTC CCA AAG ACC AC-3’; *Osterix*, 5’-AAG GGT GGG TAG TCA TTT GCA-3’ and 5’-CCC TTC TCA AGC ACC AAT GG-3’; *S100a4 (Fsp1)*, 5’-TGA GCA ACT TGG ACA GCA ACA-3’ and 5’-TTC CGG GGT TCC TTA TCT GGG-3’; *Acta2 (a-SMA)*, 5’-GTC CCA GAC ATC AGG GAG TAA-3’ and 5’-TCG GAT ACT TCA GCG TCA GGA-3’; *Col1a1*, 5’-GGC CTT GGA GGA AAC TTT GC-3’ and 5’-GGG ACC CAT TGG ACC TGA AC-3’; *Bglap (Osteocalcin),* 5’-GGA CTG AGG CTC TGT GAG GT-3’ and 5’-CAG ACA CCA TGA GGA CCA TC-3’; *Runx2*, 5’-GTT ATG AAA AAC CAA GTA GCC AGG-3’ and 5’-GTA ATC TGA CTC TGT CCT TGT GGA-3’; *Ibsp (Bsp)*, 5’-AGG ACT AGG GGT CAA ACA C-3’ and 5’-AGT AGC GTG GCC GGT ACT TA-3’; *Alpl (Tnap)*, 5’-GGG GAC ATG CAG TAT GAG TT-3’ and 5’-GGC CTG GTA GTT GTT GTG AG-3’. The relative quantification in gene expression was determined using the 2^-ΔΔCt^ method.

### RNA sequencing and analysis

Single cell suspensions were prepared from fresh tumor upon sacrifice. Tumors were minced and digested as described above. Tumor stromal cells were separated from the GFP+ tumor cells and sorted based on the expression, or lack of thereof, of TdT and CD45 markers.

Library preparation was performed with 10 ng of total RNA and integrity was determined using an Agilent bioanalyzer. ds-cDNA was prepared using the SMARTer Ultra Low RNA kit for Illumina Sequencing (Clontech) per manufacturer’s protocol. The cDNA was fragmented using a Covaris E220 sonicator using peak incident power 18, duty factor 20%, cycles/burst 50, for a 120 s. cDNA was blunt ended, had an A base added to the 3’ ends, and then had Illumina sequencing adapters ligated to the ends. Ligated fragments were then amplified for 12 cycles using primers incorporating unique index tags. Fragments were sequenced on an Illumina HiSeq 3000 using single end reads extending 50 bases. Basecalls and demultiplexing were performed with Illumina’s bcl2fastq software and a custom python demultiplexing program with a maximum of one mismatch in the indexing read. RNA-seq reads were then aligned to the *Mus musculus* Ensembl release 76 top-level assembly with STAR version 2.0.4b (Dobin et al., 2013). Gene counts were derived from the number of uniquely aligned unambiguous reads by Subread:featureCount version 1.4.5 (Liao et al., 2014). Isoform expression of known Ensembl transcripts were estimated with Sailfish version 0.6.13 (Patro et al., 2017). Sequencing performance was assessed with RSeQC version 2.3 (Wang et al., 2012).

All gene counts were then imported into the R/Bioconductor package EdgeR (Robinson et al., 2010) and TMM normalization size factors were calculated to adjust for samples for differences in library size. Ribosomal genes and genes not expressed in at least two samples greater than one count-per-million were excluded from further analysis. The TMM size factors and the matrix of counts were then imported into the R/Bioconductor package Limma (Ritchie et al., 2015) and weighted likelihoods based on the observed mean-variance relationship of every gene and sample were then calculated with Limma’s voomWithQualityWeights (Liu et al., 2015). The data were then fitted to a Limma generalized linear model to test for changes in a single cell population relative to the mean of all other populations to find only genes that were uniquely up-regulated with Benjamini-Hochberg false-discovery rate adjusted p-values less than or equal to 0.05. For each group of cells, global perturbations in known Gene Ontology (GO) terms were then measured using the R/Bioconductor package GAGE (Luo et al., 2009) to quantify the mean log two fold-changes of ll genes in a given term versus the background log two fold-changes of all genes found outside the respective term. Only globally up-regulated GO terms with Benjamini-Hochberg false-discovery rate adjusted p-values less than or equal to 0.05 were considered for comparison across populations.

## Data Availability

Sequencing data are deposited in GEO under accession code GSE143586 https://www.ncbi.nlm.nih.gov/geo/query/acc.cgi?acc=GSE143586. The following dataset was generated: FaccioRRicciBTycksenECivitelliRFontanaFCelikHBelleJI2020GSE143586NCBI Gene Expression OmnibusGSE14358610.7554/eLife.54659PMC742830632755539

## Additional files

Transparent reporting form

## References

[bib1] Abe R, Donnelly SC, Peng T, Bucala R, Metz CN (2001). Peripheral blood fibrocytes: differentiation pathway and migration to wound sites. The Journal of Immunology.

[bib2] Baek WY, Lee MA, Jung JW, Kim SY, Akiyama H, de Crombrugghe B, Kim JE (2009). Positive regulation of adult bone formation by osteoblast-specific transcription factor osterix. Journal of Bone and Mineral Research.

[bib3] Bartoschek M, Oskolkov N, Bocci M, Lövrot J, Larsson C, Sommarin M, Madsen CD, Lindgren D, Pekar G, Karlsson G, Ringnér M, Bergh J, Björklund Åsa, Pietras K (2018). Spatially and functionally distinct subclasses of breast cancer-associated fibroblasts revealed by single cell RNA sequencing. Nature Communications.

[bib4] Capietto AH, Kim S, Sanford DE, Linehan DC, Hikida M, Kumosaki T, Novack DV, Faccio R (2013). Down-regulation of PLCγ2-β-catenin pathway promotes activation and expansion of myeloid-derived suppressor cells in Cancer. The Journal of Experimental Medicine.

[bib5] Chen J, Shi Y, Regan J, Karuppaiah K, Ornitz DM, Long F (2014). Osx-Cre targets multiple cell types besides osteoblast lineage in postnatal mice. PLOS ONE.

[bib6] Chen X, Song E (2019). Turning foes to friends: targeting cancer-associated fibroblasts. Nature Reviews Drug Discovery.

[bib7] Corsa CA, Brenot A, Grither WR, Van Hove S, Loza AJ, Zhang K, Ponik SM, Liu Y, DeNardo DG, Eliceiri KW, Keely PJ, Longmore GD (2016). The action of discoidin domain receptor 2 in basal tumor cells and stromal Cancer-Associated fibroblasts is critical for breast Cancer metastasis. Cell Reports.

[bib8] Costa A, Kieffer Y, Scholer-Dahirel A, Pelon F, Bourachot B, Cardon M, Sirven P, Magagna I, Fuhrmann L, Bernard C, Bonneau C, Kondratova M, Kuperstein I, Zinovyev A, Givel AM, Parrini MC, Soumelis V, Vincent-Salomon A, Mechta-Grigoriou F (2018). Fibroblast heterogeneity and immunosuppressive environment in human breast Cancer. Cancer Cell.

[bib9] Croft AP, Campos J, Jansen K, Turner JD, Marshall J, Attar M, Savary L, Wehmeyer C, Naylor AJ, Kemble S, Begum J, Dürholz K, Perlman H, Barone F, McGettrick HM, Fearon DT, Wei K, Raychaudhuri S, Korsunsky I, Brenner MB, Coles M, Sansom SN, Filer A, Buckley CD (2019). Distinct fibroblast subsets drive inflammation and damage in arthritis. Nature.

[bib10] D'Amico L, Mahajan S, Capietto AH, Yang Z, Zamani A, Ricci B, Bumpass DB, Meyer M, Su X, Wang-Gillam A, Weilbaecher K, Stewart SA, DeNardo DG, Faccio R (2016). Dickkopf-related protein 1 (Dkk1) regulates the accumulation and function of myeloid derived suppressor cells in Cancer. Journal of Experimental Medicine.

[bib11] Davey RA, Clarke MV, Sastra S, Skinner JP, Chiang C, Anderson PH, Zajac JD (2012). Decreased body weight in young Osterix-Cre transgenic mice results in delayed cortical bone expansion and accrual. Transgenic Research.

[bib12] de Vega S, Kondo A, Suzuki M, Arai H, Jiapaer S, Sabit H, Nakada M, Ikeuchi T, Ishijima M, Arikawa-Hirasawa E, Yamada Y, Okada Y (2019). Fibulin-7 is overexpressed in glioblastomas and modulates glioblastoma neovascularization through interaction with angiopoietin-1. International Journal of Cancer.

[bib13] Dobin A, Davis CA, Schlesinger F, Drenkow J, Zaleski C, Jha S, Batut P, Chaisson M, Gingeras TR (2013). STAR: ultrafast universal RNA-seq aligner. Bioinformatics.

[bib14] Elyada E, Bolisetty M, Laise P, Flynn WF, Courtois ET, Burkhart RA, Teinor JA, Belleau P, Biffi G, Lucito MS, Sivajothi S, Armstrong TD, Engle DD, Yu KH, Hao Y, Wolfgang CL, Park Y, Preall J, Jaffee EM, Califano A, Robson P, Tuveson DA (2019). Cross-Species Single-Cell analysis of pancreatic ductal adenocarcinoma reveals Antigen-Presenting Cancer-Associated fibroblasts. Cancer Discovery.

[bib15] Engblom C, Pfirschke C, Zilionis R, Da Silva Martins J, Bos SA, Courties G, Rickelt S, Severe N, Baryawno N, Faget J, Savova V, Zemmour D, Kline J, Siwicki M, Garris C, Pucci F, Liao HW, Lin YJ, Newton A, Yaghi OK, Iwamoto Y, Tricot B, Wojtkiewicz GR, Nahrendorf M, Cortez-Retamozo V, Meylan E, Hynes RO, Demay M, Klein A, Bredella MA, Scadden DT, Weissleder R, Pittet MJ (2017). Osteoblasts remotely supply lung tumors with cancer-promoting SiglecF^high^ neutrophils. Science.

[bib16] Finak G, Bertos N, Pepin F, Sadekova S, Souleimanova M, Zhao H, Chen H, Omeroglu G, Meterissian S, Omeroglu A, Hallett M, Park M (2008). Stromal gene expression predicts clinical outcome in breast Cancer. Nature Medicine.

[bib17] Fontana F, Hickman-Brecks CL, Salazar VS, Revollo L, Abou-Ezzi G, Grimston SK, Jeong SY, Watkins M, Fortunato M, Alippe Y, Link DC, Mbalaviele G, Civitelli R (2017). N-cadherin regulation of bone growth and homeostasis is osteolineage Stage-Specific. Journal of Bone and Mineral Research.

[bib18] Gkretsi V, Stylianopoulos T (2018). Cell adhesion and matrix stiffness: coordinating Cancer cell invasion and metastasis. Frontiers in Oncology.

[bib19] González-Domínguez É, Samaniego R, Flores-Sevilla JL, Campos-Campos SF, Gómez-Campos G, Salas A, Campos-Peña V, Corbí ÁL, Sánchez-Mateos P, Sánchez-Torres C (2015). CD163L1 and CLEC5A discriminate subsets of human resident and inflammatory macrophages in vivo. Journal of Leukocyte Biology.

[bib20] Gori F, Schipani E, Demay MB (2001). Fibromodulin is expressed by both chondrocytes and osteoblasts during fetal bone development. Journal of Cellular Biochemistry.

[bib21] Goto H, Nishioka Y (2017). Fibrocytes: a novel stromal cells to regulate resistance to Anti-Angiogenic therapy and Cancer progression. International Journal of Molecular Sciences.

[bib22] Goubran HA, Kotb RR, Stakiw J, Emara ME, Burnouf T (2014). Regulation of tumor growth and metastasis: the role of tumor microenvironment. Cancer Growth and Metastasis.

[bib23] Hanahan D, Weinberg RA (2011). Hallmarks of Cancer: the next generation. Cell.

[bib24] Henson SM, Akbar AN (2009). KLRG1—more than a marker for T cell senescence. Age.

[bib25] Ikeuchi T, de Vega S, Forcinito P, Doyle AD, Amaral J, Rodriguez IR, Arikawa-Hirasawa E, Yamada Y (2018). Extracellular protein Fibulin-7 and its C-Terminal fragment have in vivo antiangiogenic activity. Scientific Reports.

[bib26] Jia Z, Wang S, He D, Cui L, Lu Y, Hu H, Qin B, Zhao Z (2015). Role of calcium in the regulation of bone morphogenetic protein 2, runt-related transcription factor 2 and osterix in primary renal tubular epithelial cells by the vitamin D receptor. Molecular Medicine Reports.

[bib27] Kalluri R, Zeisberg M (2006). Fibroblasts in cancer. Nature Reviews Cancer.

[bib28] Koch M, Laub F, Zhou P, Hahn RA, Tanaka S, Burgeson RE, Gerecke DR, Ramirez F, Gordon MK (2003). Collagen XXIV, a vertebrate fibrillar collagen with structural features of invertebrate collagens: selective expression in developing cornea and bone. The Journal of Biological Chemistry.

[bib29] Liao Y, Smyth GK, Shi W (2014). featureCounts: an efficient general purpose program for assigning sequence reads to genomic features. Bioinformatics.

[bib30] Liu Y, Strecker S, Wang L, Kronenberg MS, Wang W, Rowe DW, Maye P (2013). Osterix-cre labeled progenitor cells contribute to the formation and maintenance of the bone marrow stroma. PLOS ONE.

[bib31] Liu R, Holik AZ, Su S, Jansz N, Chen K, Leong HS, Blewitt ME, Asselin-Labat ML, Smyth GK, Ritchie ME (2015). Why weight? modelling sample and observational level variability improves power in RNA-seq analyses. Nucleic Acids Research.

[bib32] Luo W, Friedman MS, Shedden K, Hankenson KD, Woolf PJ (2009). GAGE: generally applicable gene set enrichment for pathway analysis. BMC Bioinformatics.

[bib33] Matsuo N, Tanaka S, Gordon MK, Koch M, Yoshioka H, Ramirez F (2006). CREB-AP1 protein complexes regulate transcription of the collagen XXIV gene (Col24a1) in osteoblasts. Journal of Biological Chemistry.

[bib34] McDonald LT, LaRue AC (2012). Hematopoietic stem cell derived carcinoma-associated fibroblasts: a novel origin. International Journal of Clinical and Experimental Pathology.

[bib35] Meyer MA, Baer JM, Knolhoff BL, Nywening TM, Panni RZ, Su X, Weilbaecher KN, Hawkins WG, Ma C, Fields RC, Linehan DC, Challen GA, Faccio R, Aft RL, DeNardo DG (2018). Breast and pancreatic Cancer interrupt IRF8-dependent dendritic cell development to overcome immune surveillance. Nature Communications.

[bib36] Miura Y, Ota S, Peterlin M, McDevitt G, Kanazawa S (2019). A subpopulation of synovial fibroblasts leads to osteochondrogenesis in a mouse model of chronic inflammatory rheumatoid arthritis. JBMR Plus.

[bib37] Mizoguchi T, Pinho S, Ahmed J, Kunisaki Y, Hanoun M, Mendelson A, Ono N, Kronenberg HM, Frenette PS (2014). Osterix marks distinct waves of primitive and definitive stromal progenitors during bone marrow development. Developmental Cell.

[bib38] Monterubbianesi R, Bencun M, Pagella P, Woloszyk A, Orsini G, Mitsiadis TA (2019). A comparative in vitro study of the osteogenic and adipogenic potential of human dental pulp stem cells, gingival fibroblasts and foreskin fibroblasts. Scientific Reports.

[bib39] Nakashima K, Zhou X, Kunkel G, Zhang Z, Deng JM, Behringer RR, de Crombrugghe B (2002). The novel zinc finger-containing transcription factor osterix is required for osteoblast differentiation and bone formation. Cell.

[bib40] Ohishi M, Ono W, Ono N, Khatri R, Marzia M, Baker EK, Root SH, Wilson TL, Iwamoto Y, Kronenberg HM, Aguila HL, Purton LE, Schipani E (2012). A novel population of cells expressing both hematopoietic and mesenchymal markers is present in the normal adult bone marrow and is augmented in a murine model of marrow fibrosis. The American Journal of Pathology.

[bib41] Orestes-Cardoso S, Nefussi JR, Lezot F, Oboeuf M, Pereira M, Mesbah M, Robert B, Berdal A (2002). Msx1 is a regulator of bone formation during development and postnatal growth: in vivo investigations in a transgenic mouse model. Connective Tissue Research.

[bib42] Patro R, Duggal G, Love MI, Irizarry RA, Kingsford C (2017). Salmon provides fast and bias-aware quantification of transcript expression. Nature Methods.

[bib43] Pietras EM, Reynaud D, Kang YA, Carlin D, Calero-Nieto FJ, Leavitt AD, Stuart JM, Göttgens B, Passegué E (2015). Functionally distinct subsets of Lineage-Biased multipotent progenitors control blood production in normal and regenerative conditions. Cell Stem Cell.

[bib44] Pourhanifeh MH, Mohammadi R, Noruzi S, Hosseini SA, Fanoudi S, Mohamadi Y, Hashemzehi M, Asemi Z, Mirzaei HR, Salarinia R, Mirzaei H (2019). The role of fibromodulin in Cancer pathogenesis: implications for diagnosis and therapy. Cancer Cell International.

[bib45] Raz Y, Cohen N, Shani O, Bell RE, Novitskiy SV, Abramovitz L, Levy C, Milyavsky M, Leider-Trejo L, Moses HL, Grisaru D, Erez N (2018). Bone marrow-derived fibroblasts are a functionally distinct stromal cell population in breast Cancer. Journal of Experimental Medicine.

[bib46] Rhodes DR, Yu J, Shanker K, Deshpande N, Varambally R, Ghosh D, Barrette T, Pandey A, Chinnaiyan AM (2004). ONCOMINE: a Cancer microarray database and integrated data-mining platform. Neoplasia.

[bib47] Ritchie ME, Phipson B, Wu D, Hu Y, Law CW, Shi W, Smyth GK (2015). Limma powers differential expression analyses for RNA-sequencing and microarray studies. Nucleic Acids Research.

[bib48] Robinson MD, McCarthy DJ, Smyth GK (2010). edgeR: a bioconductor package for differential expression analysis of digital gene expression data. Bioinformatics.

[bib49] Rodda SJ, McMahon AP (2006). Distinct roles for hedgehog and canonical wnt signaling in specification, differentiation and maintenance of osteoblast progenitors. Development.

[bib50] Strecker S, Fu Y, Liu Y, Maye P (2013). Generation and characterization of Osterix-Cherry reporter mice. Genesis.

[bib51] Su X, Esser AK, Amend SR, Xiang J, Xu Y, Ross MH, Fox GC, Kobayashi T, Steri V, Roomp K, Fontana F, Hurchla MA, Knolhoff BL, Meyer MA, Morgan EA, Tomasson JC, Novack JS, Zou W, Faccio R, Novack DV, Robinson SD, Teitelbaum SL, DeNardo DG, Schneider JG, Weilbaecher KN (2016). Antagonizing integrin β3 increases immunosuppression in Cancer. Cancer Research.

[bib52] Tan L, Sandrock I, Odak I, Aizenbud Y, Wilharm A, Barros-Martins J, Tabib Y, Borchers A, Amado T, Gangoda L, Herold MJ, Schmidt-Supprian M, Kisielow J, Silva-Santos B, Koenecke C, Hovav AH, Krebs C, Prinz I, Ravens S (2019). Single-Cell transcriptomics identifies the adaptation of Scart1^+^Vγ6^+^ T Cells to Skin Residency as Activated Effector Cells. Cell Reports.

[bib53] van Deventer HW, Palmieri DA, Wu QP, McCook EC, Serody JS (2013). Circulating fibrocytes prepare the lung for Cancer metastasis by recruiting Ly-6C+ monocytes via CCL2. The Journal of Immunology.

[bib54] Vissers LE, Cox TC, Maga AM, Short KM, Wiradjaja F, Janssen IM, Jehee F, Bertola D, Liu J, Yagnik G, Sekiguchi K, Kiyozumi D, van Bokhoven H, Marcelis C, Cunningham ML, Anderson PJ, Boyadjiev SA, Passos-Bueno MR, Veltman JA, Smyth I, Buckley MF, Roscioli T (2011). Heterozygous mutations of FREM1 are associated with an increased risk of isolated metopic craniosynostosis in humans and mice. PLOS Genetics.

[bib55] Wang L, Wang S, Li W (2012). RSeQC: quality control of RNA-seq experiments. Bioinformatics.

[bib56] Weilbaecher KN, Guise TA, McCauley LK (2011). Cancer to bone: a fatal attraction. Nature Reviews Cancer.

[bib57] Wolf MJ, Seleznik GM, Zeller N, Heikenwalder M (2010). The unexpected role of lymphotoxin β receptor signaling in carcinogenesis: from lymphoid tissue formation to liver and prostate Cancer development. Oncogene.

[bib58] Yao B, Wang J, Qu S, Liu Y, Jin Y, Lu J, Bao Q, Li L, Yuan H, Ma C (2019). Upregulated osterix promotes invasion and bone metastasis and predicts for a poor prognosis in breast Cancer. Cell Death & Disease.

